# Influence of the Cross-Sectional Shape and Corner Radius on the Compressive Behaviour of Concrete Columns Confined by FRP and Stirrups

**DOI:** 10.3390/polym14020341

**Published:** 2022-01-16

**Authors:** Yang Wei, Yang Xu, Gaofei Wang, Xunyu Cheng, Guofen Li

**Affiliations:** 1 College of Civil Engineering, Nanjing Forestry University, Nanjing 210037, China; yangyang12_07@163.com (Y.X.); wgf1997@njfu.edu.cn (G.W.); cxyyhjs@163.com (X.C.); lgf@njfu.edu.cn (G.L.); 2 Jiangsu Expressway Engineering Maintenance Technology Co., Ltd., Nanjing 211106, China

**Keywords:** FRP–stirrup composite-confined concrete, corner radius, cross-sectional shape, compressive behaviour, analytical modelling

## Abstract

Axial compression tests were carried out on 72 FRP (fiber reinforced polymer)–stirrup composite-confined concrete columns. Stirrups ensure the residual bearing capacity and ductility after the FRP fractures. To reduce the effect of stress concentration at the corners of the confined square-section concrete columns and improve the restraint effect, an FRP–stirrup composite-confined concrete structure with rounded corners is proposed. Different corner radii of the stirrup and outer FRP were designed, and the corner radius of the stirrup was adjusted accurately to meet the designed corner radius of the outer FRP. The cross-section of the specimens gradually changed from square to circular as the corner radius increased. The influence of the cross-sectional shape and corner radius on the compressive behaviour of FRP–stirrup composite-confined concrete was analysed. An increase in the corner radius can cause the strain distribution of the FRP to be more uniform and strengthen the restraint effect. The larger the corner radius of the specimen, the better the improvement of mechanical properties. The strength of the circular section specimen was greatly improved. In addition, the test parameters also included the FRP layers, FRP types and stirrup spacing. With the same corner radius, increasing the number of FRP layers or densifying the stirrup spacing effectively improved the mechanical properties of the specimens. Finally, a database of FRP–stirrup composite-confined concrete column test results with different corner radii was established. The general calculation models were proposed, respectively, for the peak points, ultimate points and stress–strain models that are applicable to FRP-, stirrup- and FRP–stirrup-confined concrete columns with different cross-sectional shapes under axial compression.

## 1. Introduction

Confined concrete is a classical structural subject. At present, stirrups and fibre-reinforced polymers (FRPs) are two types of common binding materials in engineering. Closely spaced stirrups or spiral stirrups are often used to provide a higher lateral binding force to core concrete, but their effect of improving the concrete bearing capacity in actual engineering is still limited [1,2]. FRPs have the advantages of a light weight, high strength, good corrosion resistance and fatigue resistance. It can be used to directly restrain concrete to improve its bearing capacity and deformation capacity. It can also be used in marine engineering to block the corrosion of chloride ions. At present, the research on the mechanical properties of FRP-confined concrete has been very sufficient. Research mainly focuses on the axial compression or eccentric compression properties of FRP-confined concrete [3,4,5,6,7,8,9], and some novel research, such as Wahid Ferdous et al. [10], studied the bending and shear behaviour of waste rubber concrete-filled FRP tubes with external flanges. In addition, FRP can also be made into FRP reinforcement with superior environmental and mechanical properties to replace ordinary reinforcement. For example, Omar Alajarmeh et al. [11] studied the behaviour of circular concrete columns reinforced with hollow composite sections and GFRP bars. However, because FRPs still have the disadvantages of brittle failure and a high cost, which greatly limit its application, the application of FRP-confined concrete structure is still limited. In recent years, many scholars have proposed the concept of “composite-confined concrete”, that is, FRPs and steel working together to restrain concrete [12,13,14,15,16,17,18,19,20,21,22,23], taking full advantage of the tensile properties of the FRPs and the excellent properties of steel. FRP stirrup-confined concrete columns use FRPs and stirrups as external restraint materials and synthesise the characteristics of both FRP restraints and stirrup restraints. While FRPs improve the bearing capacity of the structure, the transverse stirrups reduce the fractures of the FRPs to ensure the ductility of the confined concrete structure under axial compression [15].

In the axial compression tests of FRP-only, stirrup-only or FRP–stirrup composite-confined concrete columns, test variables usually include FRP thickness, FRP type, steel tube thickness, stirrup spacing and other common variables. Liao et al. [24] and Li et al. [25,26] studied the monotonic axial compressive properties of FRP-confined concrete using FRP tube thickness and type as test variables. The types of FRPs used in most studies are CFRP, GFRP and BFRP. Togay ozbakkaloglu et al. [27] studied the effect of uncommon AFRP on the axial stress–strain behaviour of FRP-confined concrete columns. Guo et al. [28] studied the effect of specimen size on the axial compressive properties of FRP-confined concrete columns. Wang et al. [29], Wei et al. [30,31], Liu et al. [32] and Sun et al. [33] conducted the axial compression test research of circular FRP–steel composite-confined concrete on the change in FRP thickness, FRP type and steel tube diameter-thickness ratio. Wei et al. [15] designed different FRP layers, FRP types and steel spirals spacing to study the axial compressive properties of circular FRP–steel spirals confined concrete. Zhou et al. [34] studied the axial compressive behaviour of novel poly-ethylene terephthalate (PET) FRP-confined FRP spiral reinforced concrete square columns with the number of PET FRP layers and the pitch of the GFRP spiral as test variables.

In addition, in the research on the axial compression performance of confined concrete, the section forms of the specimens are mainly circular or square, of which the most common is circular [35,36,37,38,39]. The axial compression properties of circular section specimens and square section specimens are quite different. Ayough et al. [40], Ding et al. [41] and Zhu et al. [42] compared the effects of polygonal-, circular- and square-shaped sections on the axial compression behaviour of concrete-filled steel tube (CFST) columns and found that the restraint efficiency of square specimens was far less than those of circular specimens; the load-bearing capacity and ductility of circular section specimens were better than those of square section specimens. Yaqub et al. [43] found that a square section confined by FRP contained some ineffective confinement areas and that the corners have a stress enhancement phenomenon, while the circular section contains completely effective confinement areas with FRP; due to the stress enhancement phenomenon at the corners of the square section specimens, FRP fracture mainly occurs at the corners. In addition, Zheng et al. [44] studied the differences in the stress–strain relationship curves of circular sections and square sections of concrete-filled FRP–stirrup double-skin tube columns under compression and found that the circular specimens showed a higher strength increase ratio and a greater slope of the stress–strain curve in the strengthening stage. In conclusion, there is a certain gap between the performance improvement efficiency of confined square-shaped concrete columns and that of confined circular-shaped concrete columns [18].

To reduce the effect of stress concentration at the corners of the confined square section concrete column and improve the restraint effect, rounding the square section’s corners is an effective technical measure. The axial compression characteristics of confined concrete columns with different corner radii are also very different. Gao et al. [45] and Li et al. [46] studied the axial compression behaviours of CFRP-confined steel-reinforced rectangular concrete columns and FRP-confined square concrete columns with different corner radii, and they concluded that by increasing the corner radius, the strength and ductility of the specimens increased, the circumferential strain distribution of FRP was more uniform than that of CFRP and the extent of damage to the core concrete was smaller. Both Li et al. [46] and Wang et al. [47] designed the range of corner radius ratio *ρ* of the specimen as 0~1 and showed that when the cross-section of confined concrete was close to circular (*ρ* = 1), the strength of the confined concrete column increased the most. Ceccato et al. [48], using axial compression testing, found that the extent of damage to concrete confined by FRP along the sides of the specimen increased gradually with a decreasing corner radius. When scholars studied the influence of the corner radius on the stress–strain curve of confined concrete, they found that as the corner radius increased, the decreasing curve of the stress–strain curve after the peak point was transformed into an increasing curve [16,45]. In addition to the corner radius, scholars have also studied the effects of FRP layers and stirrup spacing on the compressive performance of FRP–stirrup composite-confined concrete columns and found that the compressive strength of the concrete increased with an increase in the outer FRP layers or a decrease in the spacing of steel spirals [15,49,50].

Based on the current situation that there are many studies on the mechanical properties of confined concrete columns with circular sections, and relatively few studies on the mechanical properties of rectangular section specimens, the axial compression properties of FRP–stirrup composite specimens with different section shapes under compression are studied in this paper. The square specimens are the main research object. In order to improve the stress concentration at the corners of square section specimens, different corner radii of the stirrups and outer FRP were designed, and now, the research on the influence of different corner radii on the axial compression performance of FRP–stirrup composite-confined concrete specimens is limited. Thus, the corner radii are used as a test variable in this test. The cross-sections of the specimens were gradually changed from square to circular as the corner radii increased. Therefore, in addition to studying the influences of common test variables (the number of FRP layers, FRP types and stirrup spacing) on the axial compression properties of FRP–stirrup composite-confined concrete specimens, this test focused more on the influence of different corner radii and cross-sectional shapes.

At present, there are few theoretical calculation models for the load-carrying capacity of rectangular FRP–stirrup composite-confined concrete columns, and there are fewer calculation models suitable for quantitatively describing the mechanical properties of rectangular FRP–stirrup composite concrete columns with different cross-section characteristics under axial compression. Therefore, a database considering the effects of corner radii, FRP type, FRP layer number, stirrup spacing, etc., on the mechanical properties of FRP and stirrup-confined concrete is established, which ensures the parameter diversity of the newly proposed model fitting data. Then, we use the data in the database to evaluate the models of FRP–stirrup composite-confined circular concrete. Based on the constraint mechanism and test results, the effective restraint coefficient of the stirrup and the cross-sectional shape coefficient of the FRP were introduced to modify the calculation model of FRP–stirrup composite-confined circular concrete while further considering the reduction in the restraint effect of a small corner radius on the core concrete. Therefore, the peak points, ultimate points and stress–strain curve models considering the restraint effect of stirrups and suitable for FRP-only, stirrup-only and FRP–stirrup composite-confined concrete specimens with small corner radii and large corner radii are proposed. The proposed models provide a reference for the follow-up study of composite-confined concrete columns. Moreover, the FRP stirrup composite-confined concrete structure proposed in this paper also has certain practical significance. The composite restraint of FRP and stirrup on the concrete column makes it have a higher bearing capacity and better deformation capacity, which is of great significance to the development of various civil buildings, highways, bridges and underground structures. Due to the corrosion resistance of FRP, this structure can also be used in marine engineering to block the corrosion of chloride ions in seawater and sea sand.

## 2. Test Investigation

### 2.1. Parameters and Materials

**Figure 1:**
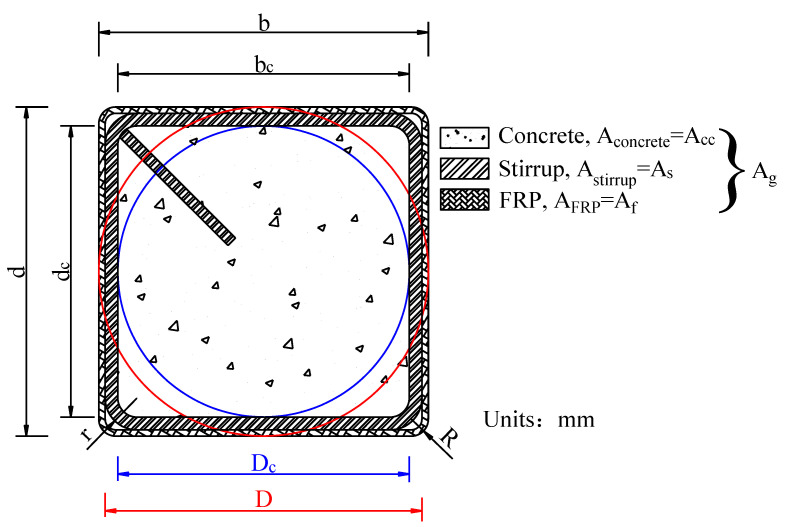
Details of FRP–steel-confined rectangular concrete columns (units: mm).

**Figure 2:**
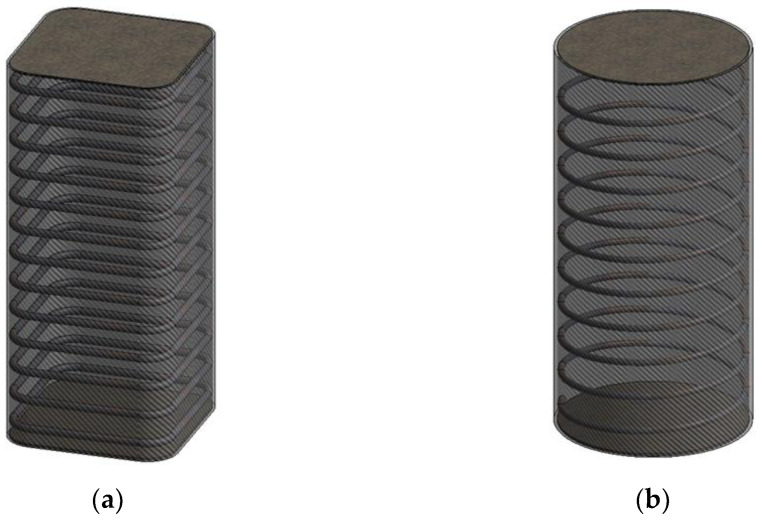
Three-dimensional schematic diagrams of specimen structures with different cross-section shapes: (**a**) square with rounded corners specimens; (**b**) circular specimens.

Seventy-two FRP–stirrup composite-confined concrete columns with heights of 300 mm were designed and tested under axial compression. Taking into account the stress concentration phenomenon of the square columns, the corners of the stirrups and outer FRP of all specimens were rounded. The corner radii of the outer FRP (R) are 5 mm, 25 mm, 40 mm and 75 mm. Because when stirrups are used to restrain concrete columns, the shape design of stirrups is usually consistent with the cross-sectional shape of specimens, so the corner radii of the stirrups (r) are adjusted accurately to meet the corner radius of the outer FRP. Due to the limitations of stirrup bending, the minimum corner radius of the stirrups could only be 15 mm. The cross-sections of the specimens gradually changed from square to circular as the corner radii increased. The length (b) and width (d) of the specimens with square sections are equal, both of which are 150 mm, and the diameter of the circular section was 150 mm. The main function of FRPs and stirrups in the structure is to serve as the external restraint material of core concrete, limit the transverse deformation of concrete under axial compression and make the concrete under three-dimensional compression so as to improve its bearing capacity and deformation capacity. Therefore, twelve unreinforced concrete specimens were constructed, consisting of six unreinforced circular columns a (φ150 mm × 300 mm (PC-C series)) and six unreinforced square columns (150 mm × 150 mm × 300 mm (PC-S series)). According to the type of external FRP (CFRP or BFRP), the specimens were divided into two batches. Each batch included seven groups of concrete specimens. The specimens were coded according to their cross-sectional shape (S: square), corner radius (R: 5, 25, 40, and 75 mm), stirrup spacing (S: 20, or 40 mm), FRP type (B: BFRP, and C: CFRP) and the number of FRP layers (0, 1 or 2 layers). Two identical specimens with the same parameters were produced. The S20 restrained concrete specimens were divided into four groups according to the corner radius of the specimens (R: 5, 25, 40, or 75 mm), and only two S40groups of specimens with corner radii (R: 25, 40 mm) were produced. To ensure the accuracy of the test, two specimens with the same parameters were prepared. The corner radii, number of FRP layers, stirrup spacing and FRP type of different specimens are shown in Table 1. Figure 1 illustrates the details of FRP–stirrup-confined concrete columns with different corner radii. Figure 2 presents three-dimensional schematic diagrams of the specimen structures with different cross-section shapes.

The concrete columns confined by BFRP and CFRP were cast in two batches with a 0.46 water-cement ratio. The cement, water, sand and gravel in the concrete were 402, 185, 598 and 1215 kg/m^3^, respectively. At the same time, six concrete cubes of 150 mm × 150 mm × 150 mm were prepared for both batches. According to the standard for test methods of mechanical properties on ordinary concrete (GB/T50081-2016) [51], the average compressive strengths of 28d concrete cube were 48.85 MPa and 36.70 MPa, respectively. There were no longitudinal reinforcements in the concrete columns, and the clear concrete cover was 0 mm for all specimens. The transverse steel reinforcement was made of eight mm diameter stirrups had a 20 or 40 mm spacing. According to the method specified in the tensile test of metallic materials (GB/T228.1-2010) [52], the average stirrup yield stress *f_y_,* yield strain *ε_y_* and modulus of elasticity *E_y_* were 325.5 MPa, 0.0024, and 201.0 GPa, respectively. The BFRP and CFRP both had the same nominal thickness of 0.167 mm. The CFRP used is produced by Nanjing mankat Technology Co., Ltd. (Nanjing, China), the BFRP is produced by Zhejiang Shijin Basalt Fiber Co., Ltd. (Zhejiang, China). According to the test method for tensile properties of orientation fibre reinforced polymer matrix composite materials (GB/T 3354-2014) [53], the mechanical properties of the FRP sheets were obtained: the average tensile strength *f_fu_*, ultimate tensile strain *ε_fu_* and elastic modulus *E_f_* were 4113 MPa, 0.0222 and 74.04 GPa for the BFRP sheets and 4334.64 MPa, 0.0175 and 249.71 GPa for the CFRP sheets, respectively. The average tensile strength, modulus of elasticity and ultimate tensile strain of the epoxy resin adhesives (L-500 AS/L-500 BS) provided by Sanyu Resin Co., Ltd. (Shanghai, China).were 67.7 MPa, 2.9 GPa and 0.029, respectively. These properties were taken from the specifications of the manufacturer.

### 2.2. Construction of the Specimens

**Figure 3:**
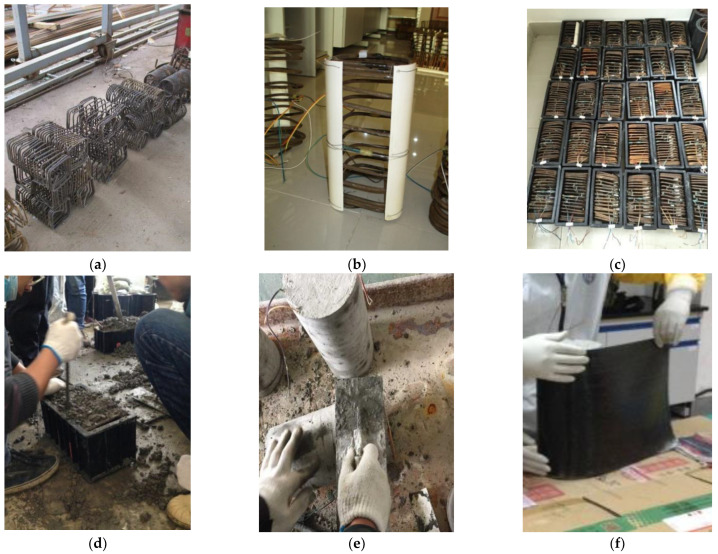
Preparation processes of rectangular concrete columns confined by FRPs and stirrups: (**a**) steel spirals; (**b**) corner treatment; (**c**) placing stirrup; (**d**) casting concrete; (**e**) polishing surface; (**f**) wrapping frp sheets.

Figure 3 presents the preparation of the FRP–stirrup composite-confined concrete columns with different corner radii. In the first step, the rectangular spiral stirrups were formed. Each rectangular spiral stirrup was made on a steel bending machine to ensure that the outer size of the section was 150 mm × 150 mm, and the corner radii of the stirrups were accurately adjusted to meet the specified value. Before the concrete columns were cast, the corner preparation of the mould and installation of the rectangular spiral stirrups were carried out. A PVC tube with a diameter of twice that of the corner radius of the concrete column was cut into four pieces along the longitudinal axis. Each PVC segment was bonded into the four corners of the rectangular mould at the same time the stirrup was placed in it. After the concrete was cast in the moulds and then cured for 28 days, the surface preparation procedure, including sanding and cleaning of concrete columns, was carried out. Finally, the surfaces of the specimens were continuously jacketed with FRP sheets with a circumferential FRP lap of 50 mm. In addition, to avoid damage, two additional 30 mm wide layers of FRP were applied at the upper and lower ends of the specimens.

### 2.3. Test Setup and Loading

**Figure 4:**
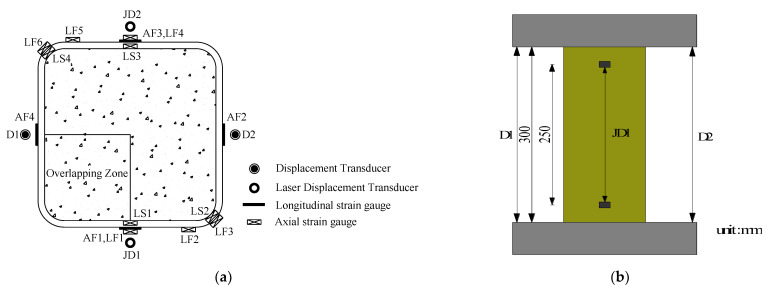
Instrumentation configuration and test setup: (**a**) layout of strain gauge and displacement transducer; (**b**) test setup.

The tests were carried out on a high-stiffness testing machine with a capacity of 3000 kN provided by Popwil Electromechanical Control Engineering Co., Ltd. (Hangzhou, China). The displacement control loading mode was adopted in the experiment. The initial loading rate was 0.3 mm/min. After FRP fracture, the loading rate increased to 0.5 mm/min. The axial loads of concrete specimens were measured by load sensors attached to the testing machine. The displacements in the whole range of specimens were measured by two common displacement meters (D) arranged symmetrically, and the displacements in the 250 mm range in the middle of the specimens were measured by two laser displacement meters (JD) arranged symmetrically. Meanwhile, the axial and longitudinal strains of the concrete specimens were measured by 5 mm × 3 mm axial strain gauges (AFs) and 100 mm × 2 mm longitudinal strain gauges (LFs) bonded onto the surfaces of the specimens. The strain data was acquired from a TDS-530 strain gauge. The instrumentation configuration and test setup for FRP–stirrup composite-confined concrete columns are shown in Figure 4.

## 3. Test Results

### 3.1. Failure Modes

**Figure 5:**
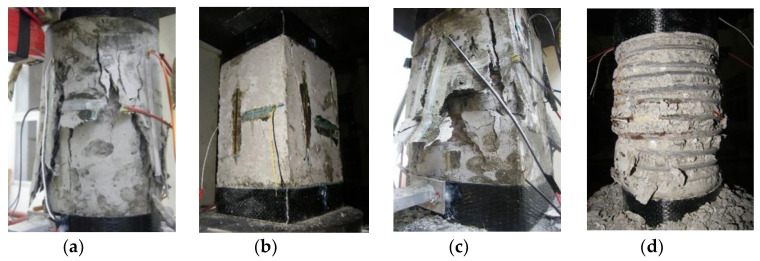

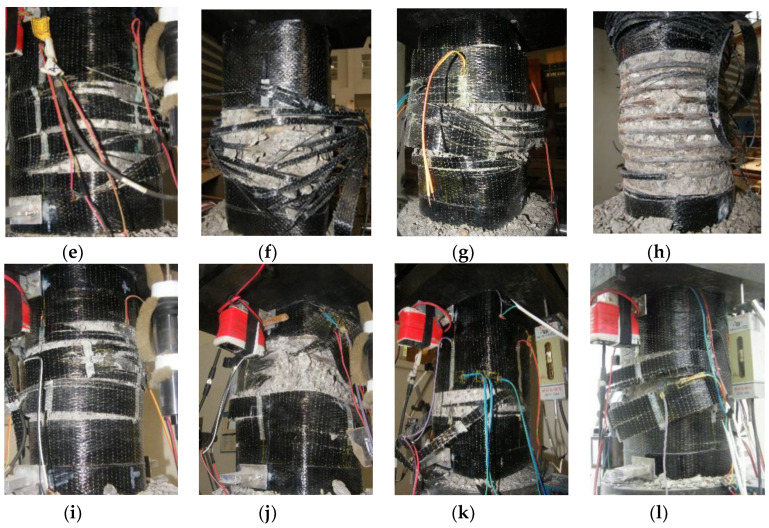
Typical failure modes of the specimens: (**a**) PC-BC; (**b**) PC-CS; (**c**) SR5S20BN; (**d**) SR75S20CN; (**e**) SR25S20B2; (**f**) SR25S20C1; (**g**) SR40S20B2; (**h**) SR75S20C2; (**i**) SR75S20B2; (**j**) SR25S40B1; (**k**) SR25S40C2; (**l**) SR40S40B2.

The typical failure modes of concrete column specimens are shown in Figure 5. The failure modes of the plain concrete column specimens (PC-C series and PC-S series) were basically the same under the axial compression tests. As there was no lateral restraint, with the increase in axial load, the surface of the plain concrete column first exhibited vertical small cracks, and the cracks on the surface of concrete continuously developed into vertical cracks along the direction of the column body. After the peak load, lateral expansion deformation occurred in the middle of the concrete column, which protruded outward. The concrete fragmentation fell in a block shape.

For the concrete columns confined only by stirrups, the corner radii of the stirrups were 15 mm, 25 mm, 40 mm and 75 mm, the cross-section gradually changed from square (R = 5 mm) to circular (R = 75 mm), and the cross-section with a corner radius of 75 mm was circular. The final failure mode of concrete columns confined only by stirrups was that almost all the concrete on the surface of the specimen fell off, and the internal stirrup was exposed. The specific failure process is as follows: when the axial load increased, small cracks appeared on the surface of the concrete, especially at the corners. When approaching the ultimate load, the external concrete was severely fragmented, and more small pieces of concrete peeled off. When the concrete column specimens were finally destroyed, nearly all the concrete on the surface fell off, and the inner stirrups were exposed. Different corner radii and stirrup spacing have a greater impact on the failure mode of concrete specimens confined only by stirrups. The increase in corner radius reduced the extent of the damage to concrete. For specimens with larger corner radii (R = 40 mm and 75 mm), the crushed concrete block was small, and the exposed area of the stirrups was less than those of specimens with smaller corner radii (R = 5 mm and 25 mm), which reflects the excellent bearing capacity of the specimens with larger corner radii. In addition, for specimens with a smaller stirrup spacing (S = 20 mm), the concrete on the surface of the specimens almost completely fell off, the core concrete still retained a good bearing capacity in the later stage of the test, showing a high ductility of the column; for specimens with a larger stirrup spacing (S = 40 mm), the concrete on the surface of the specimen will fell off completely, and the concrete between the transverse stirrups peeled off extensively, resulting in taper-shaped damage.

For the concrete columns confined by stirrups and FRP, the corner radii of the outer FRP were 5, 25, 40 and 75 mm, and the corner radii of the stirrups were adjusted accurately to meet the designed corner radius of the outer FRP. Different corner radii had obvious effects on the failure mode of FRP–stirrup composite-confined concrete columns under axial compression. At the beginning of loading, the surface of the specimen had no obvious change, and with the increase in axial load, the FRP–stirrup composite concrete columns appeared local buckling in the middle area, accompanied by a slight sound of epoxy resin glue cracking. For specimens with small corner radii, the local buckling in the middle of this specimen was more serious. Near the ultimate load, the FRP fractured. Due to the stress concentration, FRP tensile fracture mainly occurred at the corners of the concrete columns firstly. Then, with the increase in load, the fracture of FRP spread to the column body along the crack at the corner. Especially for specimens with smaller corner radii (R = 5, 25 mm), the fracture of FRP at the corner was very obvious (as shown in Figure 5f,j). For the specimens with larger corner radii (R = 40, 75 mm), the FRP exhibited tensile rupture failure; rupture failure occurred not only at the corner but also along the side of the concrete. Compared with the specimens with smaller corner radii, the specimens with larger corner radii had larger FRP fracture widths. After FRP fractured, the concrete peeled off and the stirrup was exposed, the increases of the corner radii caused the circumferential strain distributions of the FRP specimens to be more uniform and the restraint effect of the FRP to be stronger, so the crushed blocks of concrete were smaller. Additionally, when the specimen is at the later stage of axial compression, only stirrups sustain the compressive load to ensure the integrity and ductility of the specimen.

In addition, the stirrup spacing, the FRP type and the number of FRP layers also have a great influence on the failure mode of the specimens. When the built-in stirrup spacings were relatively large, the FRP fracture was obvious, and the surface concrete spalling phenomenon was more serious. The FRP fractures of the specimens wrapped with two FRP layers were mainly concentrated in the middle areas, and the fracture areas were smaller than those of the specimens wrapped with one FRP layer, but the fractures were more intense than those of the one FRP layer specimens. In the whole process of FRP fracture, the CFRP fractures were rapid, BFRP fractures lasted longer, and their failure processes were calmer.

### 3.2. Stress–Strain Response

**Figure 6:**
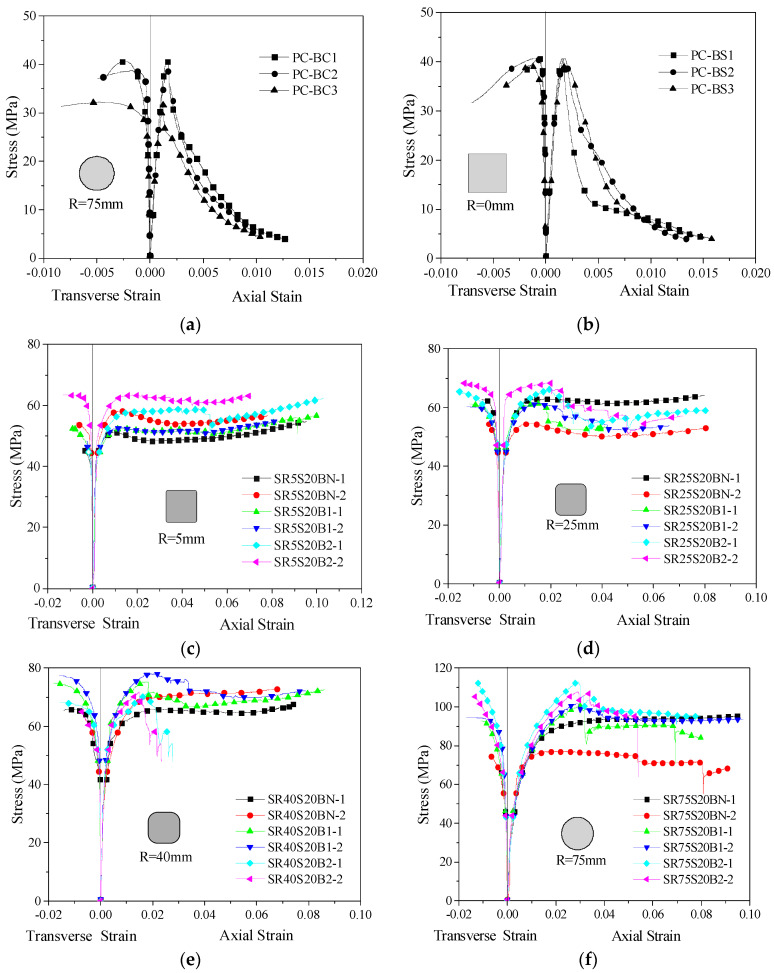

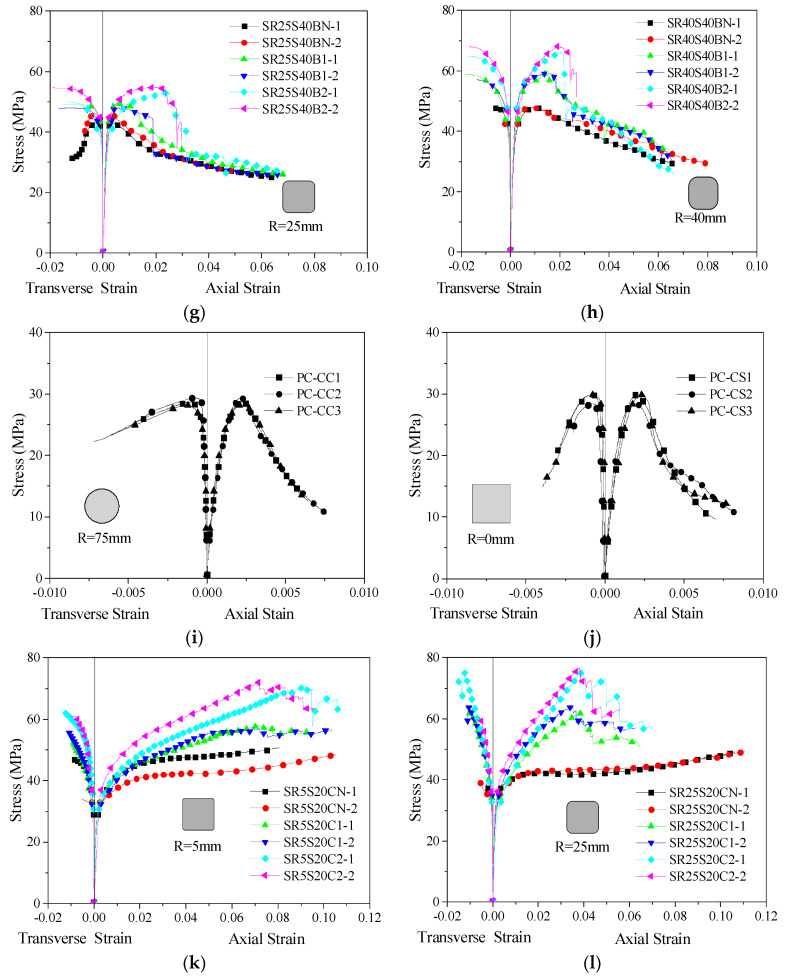

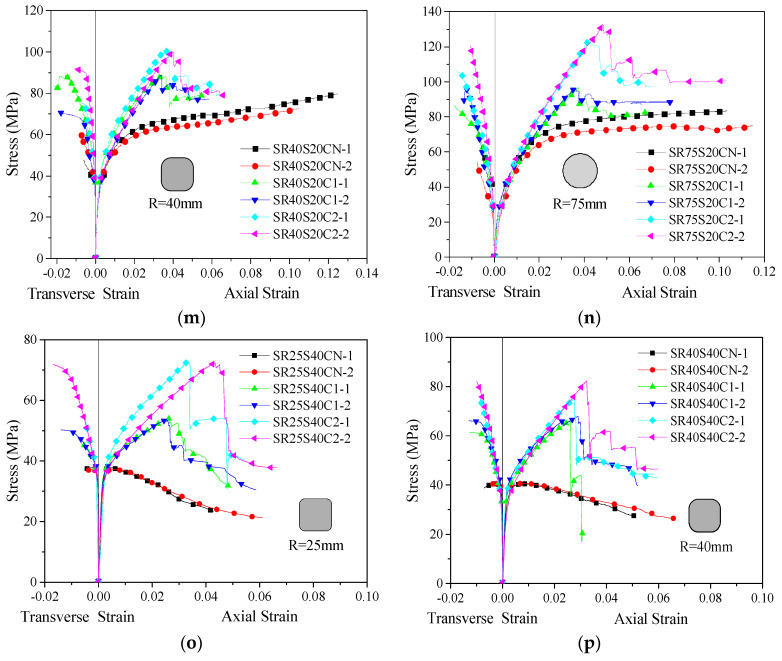
Stress-strain relationship curves of the specimens: (**a**) PC-BC; (**b**) PC-BS; (**c**) SR5S20B; (**d**) SR25S20B; (**e**) SR40S20B; (**f**) SR75S20B; (**g**) SR25S40B; (**h**) SR40S40B; (**i**) PC-CC; (**j**) PC-CS; (**k**) SR5S20C; (**l**) SR25S20C; (**m**) SR40S20C; (**n**) SR75S20C; (**o**) SR25S40C; (**p**) SR40S40C.

The stress–strain relationship curves of the concrete column specimens are given in Figure 6. The *Y*-axis is the stress, which is the ratio of the axial load to the cross-sectional area of the specimen; the *X*-axis includes the longitudinal strain and the transverse strain. The longitudinal strain is the ratio of deformation in the range of gauges measured by a laser displacement meter over the standard distance of 250 mm and corrected by comparison with the longitudinal strain gauge data. The transverse strain was obtained directly by the transverse strain gauge.

For the stirrup-only confined concrete columns, the lateral restraint force was only provided by the stirrups. The stress–strain relationship curve can be divided into three stages: the elastic stage, transition stage and stable stage. With the increase in the corner radii of the specimens, the turning points of the transition stage became more backward, showing larger ultimate stress values. After reaching the turning points of the transition stage, the bearing capacities of the specimens with dense stirrup spacing became stable, and the stress–strain relationship curves even had slight upward trends, showing the great deformation capacity and ductility of the specimens. For the specimens with sparse stirrup spacings, when the bearing capacity of the specimens reached the ultimate point, it decreased with increasing deformation, and there were no upward trends.

The stress–strain relationship curves of FRP–stirrup composite-confined concrete columns with different corner radii can be roughly divided into four stages. At the beginning of the test, the stress–strain relationship curves of the confined concrete column were basically consistent with those of the plain concrete specimens. The stress grew slowly. The concrete columns were in the stage of elastic deformation. When the axial stress reached the peak stress of the plain concrete column, the transverse deformation of the specimens increased. The outer FRP and the built-in stirrups begin to play a role in providing lateral restraint to the core concrete as the curves entered the transitional stage. With increasing axial stress, the lateral restraint force of the stirrups on the core concrete is basically constant, and the strain on the FRP increased continuously, which allowed the lateral restraint force to grow continuously, as the curves entered the strengthening stage. When the FRP broke, the loads reached their maximum values and then decreased sharply. After FRP fractured, the curve entered the residual stage, in which only the stirrups remain in the restraint working state, which ensured the ductility of the specimens.

**Figure 7:**
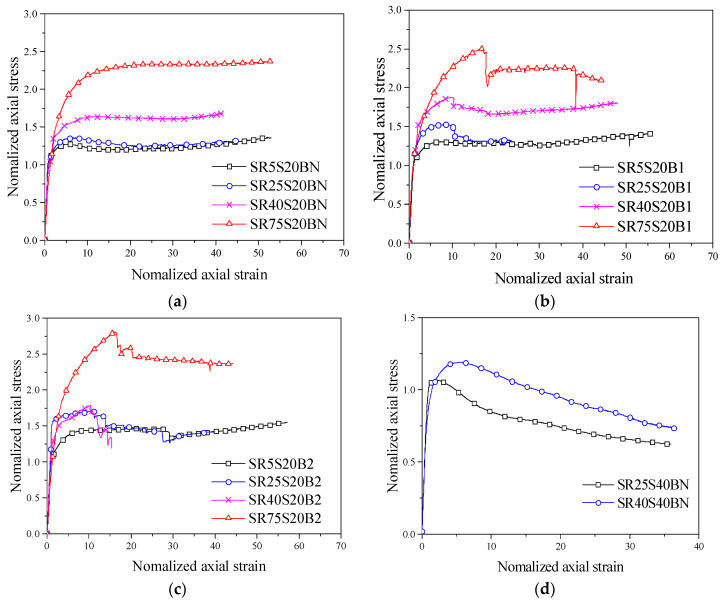

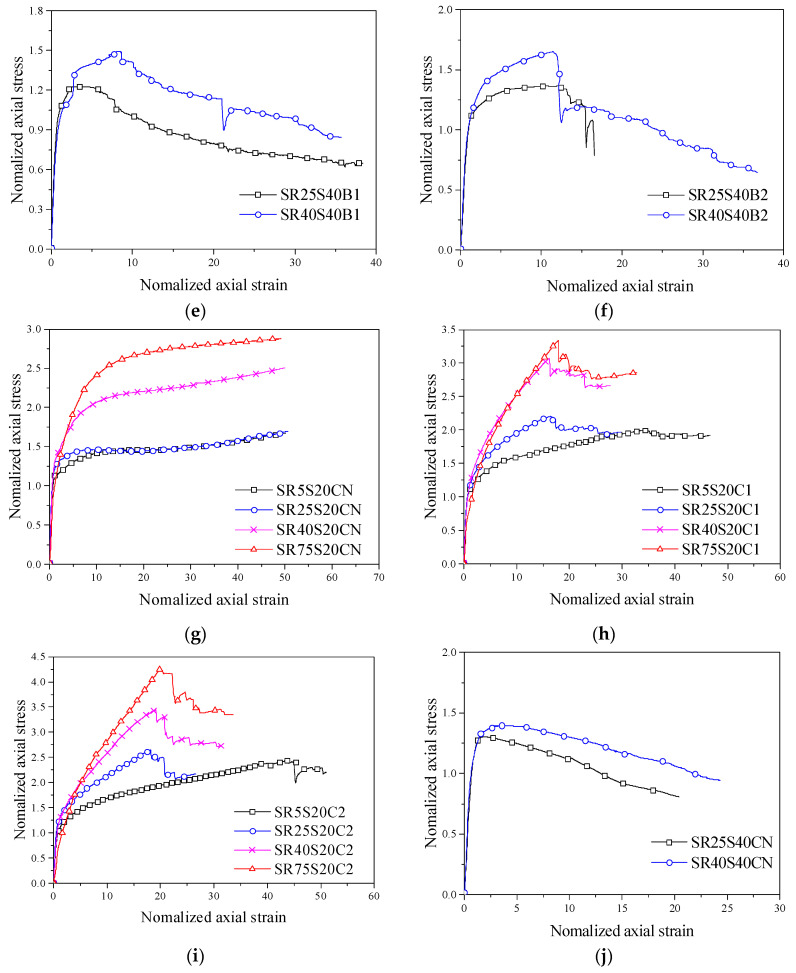

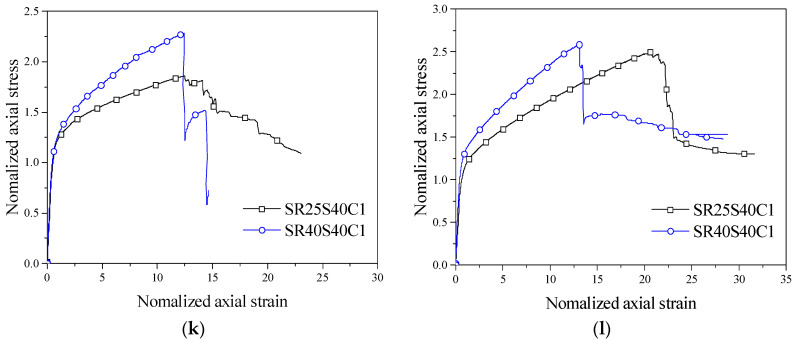
Effect of the corner radius on the stress–strain relationship curves: (**a**) S20BN series; (**b**) S20B1 series; (**c**) S20B2 series; (**d**) S40BN series; (**e**) S40B1 series; (**f**) S40B2 series; (**g**) S20CN series; (**h**) S20C1 series; (**i**) S20C2 series; (**j**) S40CN series; (**k**) S40C1 series; (**l**) S40C2 series.

To illustrate the influence of the cross-sectional shape and corner radius on the stress–strain relationship curves, the normalised stress–strain relationship curves of stirrup-only and FRP–stirrup composite-confined concrete columns are compared in Figure 7. The *Y*-axis is the normalised axial stress *f_cu_*/*f_co_*, which is the ratio of the axial stress of the specimen to the strength of unconfined concrete; the *X*-axis is the normalised axial strain of the specimen *ε_cu_*/*ε_co_*, which is the ratio of the longitudinal strain of the specimen to the strain of unconfined concrete. As presented in Figure 7, the slopes (secondary stiffness) of the FRP stirrup composite-confined concrete specimens in the strengthening stage were greatly affected by the corner radii. For the confined concrete specimens with the smallest corner radius (R = 5 mm), the stress–strain relationship curve in the strengthening stage is not obvious because it is almost a stable straight line, indicating that the specimen with a nearly square section makes the restraint distribution of FRP uneven and the constraint effect weakened. The larger the corner radius, the greater the slope and stiffness of the specimen in the strengthening stage; the more backwards the peak point and the ultimate point on the curve, indicating that the stronger the ultimate bearing capacity and deformation capacity of the specimen. Especially for the specimens with circular sections (R = 75 mm), the longitudinal stress distribution and the circumferential strain distribution of FRP were uniform, so the curve of the strengthening stage was the steepest and the duration of the strengthening stage was the longest, showing their optimal mechanical properties. Meanwhile, the larger the corner radius of the specimen was, the larger the residual bearing capacity.

In addition, the spacing of the built-in stirrups also had a great influence on the residual stage of the curves. The stress–strain relationship curves of the specimens with dense stirrup spacing, such as SR5S20B1, SR25S20B2, SR5S20C1 and SR25S20C2, show upward trends in the residual stage, and the stress value drops of specimens SR75S20B1 and SR75S20C2 in the residual stage were small, showing the excellent ductility of the specimens.

### 3.3. Influence of Parameters on the Compressive Behaviour of Confined Concrete

**Figure 8:**
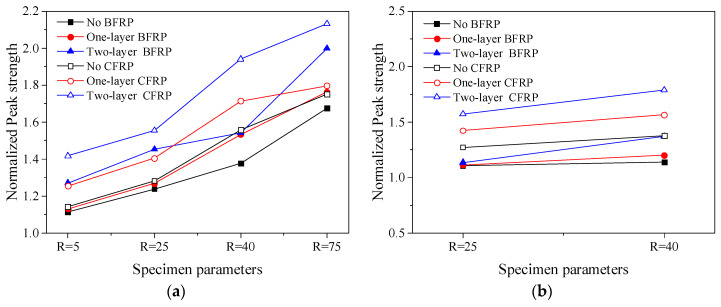

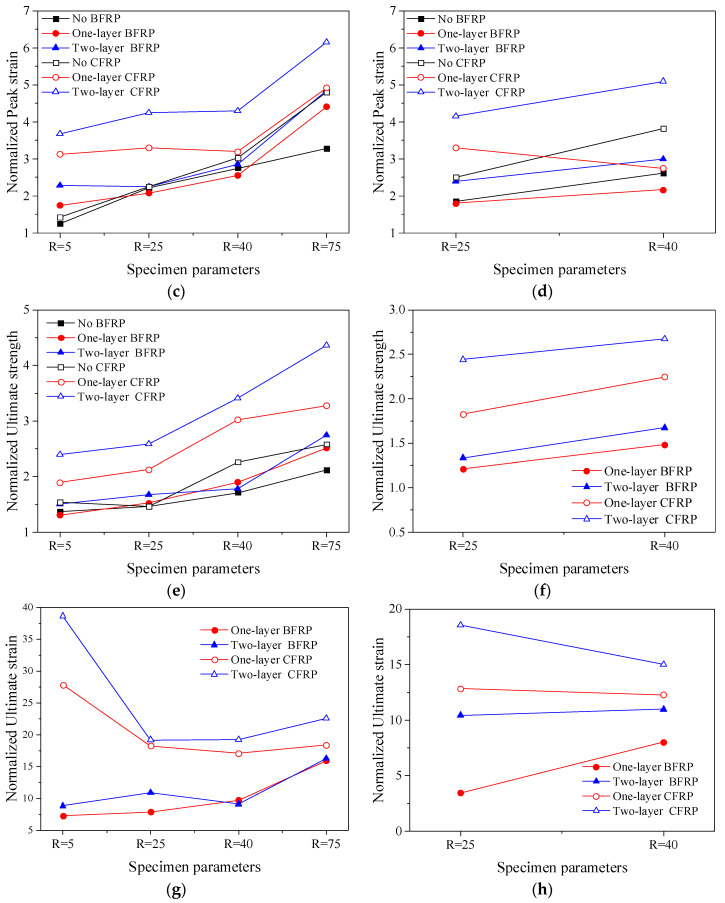
Comparison of the performance of FRP–stirrup-confined rectangular concrete columns: (**a**) peak stress (S20); (**b**) peak stress (S40); (**c**) peak strain (S20); (**d**) peak strain (S40); (**e**) ultimate stress (S20); (**f**) ultimate stress (S40); (**g**) ultimate strain (S20); (**h**) ultimate strain (S40).

**Figure 9:**
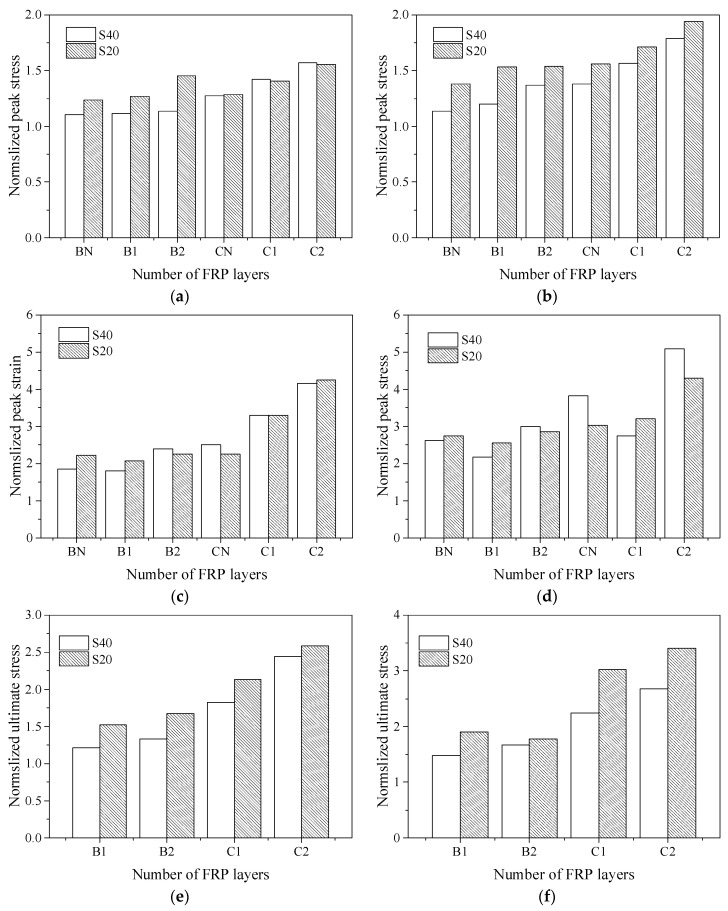

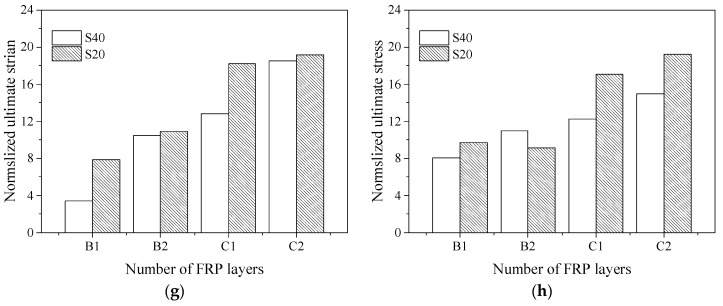
Effect of the stirrup spacing on the compressive behaviour of confined concrete columns: (**a**) peak stress (R25); (**b**) peak stress (R40); (**c**) peak strain (R25); (**d**) peak strain (R40); (**e**) ultimate stress (R25); (**f**) ultimate stress (R40); (**g**) ultimate strain (R20); (**h**) ultimate strain (R40).

The influence of the test parameters on the compressive behaviour of confined concrete columns is compared in Figure 8 and Figure 9. The *X*-axis is the corner radius of the outer FRP and the number of FRP layers, and the *Y*-axis is the ratio of the peak stress, peak strain, ultimate stress and ultimate strain to the corresponding values of the plain concrete column, respectively. In addition, the values are the averages of the two specimens in each group in the figure.

When the FRP layers, stirrup spacing and FRP type are the same, the larger the corner radius of the cross-section, the better the improvement of the mechanical properties of FRP–stirrup composite-confined concrete specimens. When the stirrup spacings were 20 mm, compared with the ultimate stress and the ultimate strain of plain concrete columns, with the increases of corner radius of outer FRP (R = 5, 25, 40, 75 mm), the peak stress, peak strain, ultimate stress and ultimate strain of specimens confined by two layers of CFRP increased by 1.57 to 2.41 times, 6.13 to 7.68 times, 2.42 to 4.34 times and 19.15 to 22.65 times, respectively; the peak stress, peak strain, ultimate stress and ultimate strain of specimens confined by one layer of BFRP increased by 1.13 to 1.76 times, 1.75 to 4.42 times, 1.31 to 2.52 times and 7.28 to 15.94 times, respectively. When the stirrup spacing was 40 mm, compared with the ultimate stress and the ultimate strain of plain concrete columns, and when the corner radius of the test piece increases from 25 mm to 40 mm, the peak stress, peak strain and ultimate stress of specimens confined by two layers of CFRP increased by 13.7%, 22.9% and 9.5%, respectively; the peak stress, peak strain, ultimate stress and ultimate strain of specimens confined by two layers of BFRP increased by 20.6%, 25.6%, 25.4% and 5.3%, respectively. In particular, when the radius of the corner was very small (5 mm), the confinement effect of FRP was the worst, and the slope of the stress–strain relationship curve was very small in the strengthening stage. At this time, the confinement pressure of FRP was small. Since FRP does not easily fracture, the ultimate strain of the specimen under compression will be the largest, as presented in Figure 8g,h. The setting of the section corner can effectively alleviate the stress concentration in the corners of the confined rectangular concrete column. The larger the corner radius is, the closer the section approaches the circular section, the more uniform the lateral expansion deformation of concrete was, and the more effective the lateral restraint provided by FRP. In conclusion, a larger corner radius can obtain larger ultimate stress and strain values.

With the same corner radius and FRP layers, the smaller the stirrup spacing, the better the achievable bearing capacity and deformation capacity, as presented in Figure 9. For example, the ultimate stress and strain of SR40S20C2 with a stirrup spacing of 20 mm were 27.5% and 28.4% higher than those of SR40S40C1 with a spacing of 40 mm. According to the results of the FRP tensile tests, in the cases with the same number of FRP layers, CFRP had larger confinement strength and confinement stiffness values. For example, the ultimate stress and ultimate strain of SR25S40C2 were 33.8% and 97.4%, respectively, higher than those of SR25S40B2. In addition, increasing the number of FRP layers can effectively improve the bearing capacity and deformation capacity of the specimens under the same corner radius and stirrup spacing of the specimen, as presented in Figure 9. For example, the ultimate stress and ultimate strain of SR25S20B2 were 9.6% and 38.5% higher than those of SR25S20B1, respectively.

## 4. Existing Models

### 4.1. Effective Confinement Pressure

**Figure 10:**
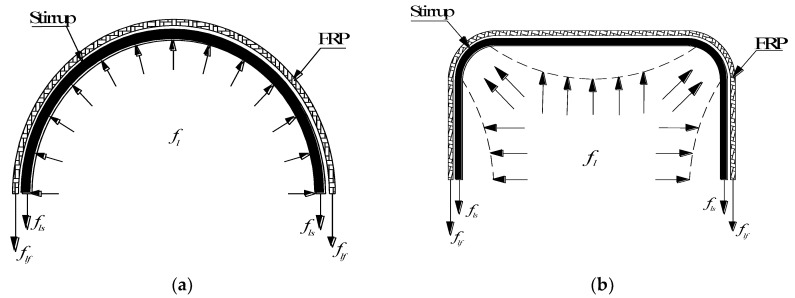
Forces on the FRP–stirrup composite-confined concrete specimens: (**a**) circular section; (**b**) rectangular section.

The lateral restraint strength *f_l_* is an important factor affecting the mechanical properties of confined concrete. It refers to the lateral restraint of the confining material on the core concrete columns, which directly determines the restraint efficiency of confined concrete structures. Figure 10 shows the forces on the concrete specimens confined by FRP and stirrups. The lateral restraints on concrete cylinders are uniform and continuous. The formula for calculating the coupling lateral restraint strength *f_l_* of FRP and stirrups on circular concrete columns is shown in Equation (1), in which *A_g_* is the total section area of restrained concrete specimens (including core concrete cross-sectional area *A_cc_*, stirrup cross-sectional area *A_s_* and FRP cross-sectional area *A_f_*), *f_lf_* is the effective lateral restraint force of the FRP and *f_ls_* is the effective lateral restraint force of the spiral stirrups. The area distribution of each component of the specimen is shown in Figure 1.

(1) $$f_{l}=f_{lf}+f_{ls}\frac{A_{cc}}{A_{g}}$$

For FRP–stirrup composite-confined rectangular concrete columns, the confinement forces at the corners of the section are larger than that at the middle of the side of the confined concrete columns. Considering the influence of a nonuniform confinement force in the calculation and analysis of rectangular concrete columns confined by FRP and stirrups is difficult. The concept of an effective lateral confinement force has been widely used in the study of confined concrete.

Mander et al. [54] calculated the effective lateral restraint force *f_ls_* of rectangular spiral stirrups on the core concrete by introducing the effective restraint coefficient *k_e_* of the stirrups on the basis of experiments and considering the influence of rectangular section characteristics on core restraint concrete.

(2) $$f_{ls}=k_{e}f_{l}^{\prime }$$

(3) $$k_{e}=\frac{\left(1-\sum_{i=1}^{n}\frac{{\left(w_{i}\right)}^{2}}{6b_{c}d_{c}}\right)\left(1-\frac{s^{\prime }}{2b_{c}}\right)\left(1-\frac{s^{\prime }}{2d_{c}}\right)}{\left(1-\rho_{cc}\right)}$$

(4) $$f_{l}^{\prime }=\frac{A_{s}}{sd_{c}}f_{y}\;\mathrm{or}\;f_{l}^{\prime }=\frac{A_{s}}{sb_{c}}f_{y}$$

where *ρ_cc_* is the volume ratio of the longitudinal reinforcement; *w_i_* is the distance between adjacent longitudinal bars; *n* is the number of longitudinal bars; *b_c_* and *d_c_* are the length and width of the core concrete, respectively (*d_c_* ≥ *b_c_*); *s* is the spacing between adjacent stirrups; *s’* is the vertical distance in the concrete between adjacent stirrups; *A_s_* is the cross-sectional area of the stirrup; and *f_y_* is the average stirrup yield stress.

Lam and Teng [55] defined the section shape factor *k_s_* of FRP-confined rectangular concrete columns with different cross-sectional shapes and calculated the effective lateral restraint force *f_lf_* of FRP:

(5) $$f_{lf}=k_{s}f_{l}=k_{s}\frac{2E_{frp}\varepsilon_{frp}t}{D}$$

(6) $$k_{s}={\left(\frac{b}{d}\right)}^{2}\frac{1-\left[\left(b/d\right){\left(d-2R\right)}^{2}+\left(d/b\right){\left(b-2R\right)}^{2}\right]}{\left(1-\rho_{cc}\right)\left(3A_{g}-\rho_{cc}\right)}$$

(7) $$D=\frac{2bd}{b+d}$$

(8) $$A_{g}=bd-(4-\pi )R^{2}$$

where *E_frp_* is the elastic modulus of the FRP; *ε_frp_* is the ultimate tensile strain of the FRP; *t* is the thickness of the FRP; *D* is the equivalent cylinder diameter; *b* and *d* are the length and width of the rectangular section, respectively; and *R* is the corner radii of the outer FRP.

### 4.2. Confinement Models

The existing stress–strain relationship models of FRP–stirrup composite-confined concrete columns have been summarised, as presented in Table 2. For the convenience of discussion, some common parameters in the following model calculation formulas are unified, and their symbolic meanings are as follows. *f_co_* and *ε_co_* are the peak stress and corresponding strain of plain concrete, respectively; *b*, *d* and *R* are the length, width and corner radius of the cross-section, respectively; *ρ_f_*, *ε_f_* and *E_f_* are the content, ultimate strain and elastic modulus of FRPs, respectively; *Ρ_s_* and *E_s_* are volume ratio and elastic modulus of stirrup, respectively; *f_lf,e_*, *f_ls,e_* and *f_l,e_* are the effective lateral restraint strength of the FRP, stirrup and the whole structure, respectively. Most of the models are suitable for circular or rectangular cross-sections, and a few are suitable for both common rectangular and circular sections and rectangular sections with rounded corners.

## 5. Proposed Analytical Model

### 5.1. Test Database

To establish a widely applicable model of FRP–stirrup composite-confined concrete columns with different corner radii, a database including the test results of FRP–stirrup composite-confined concrete columns with different corner radii by Harajli et al. [56], Ilki et al. [58], Eid et al. [61] and Paula et al. [62] and the test results of FRP-confined concrete with different corner radii by Lam and Teng [55] and Wang and Wu [47] was established. A total of 134 test data points of confined concrete with different corner radii are available, including 24 FRP-only confined concrete columns, 24 stirrup-only confined concrete columns and 86 FRP–stirrup composite-confined concrete columns. The cross-sections of the specimens in the test database gradually change from square to circular. The restraint efficiency statistics of the confined concrete columns for each study are shown in Table 3.

### 5.2. Stress–Strain Relationship Modelling

Most of the existing stress–strain models of FRP–stirrup composite-confined concrete columns only consider the change in the stress–strain relationship curve before the concrete column structure reaches its ultimate bearing capacity, and only a few stress–strain models can be used for predicting the descending curve after FRP fracture. In order to study the constitutive model of FRP stirrup composite-confined rectangular concrete column under axial compression under multi-parameter conditions, Wu and Wei [63] innovatively introduced parameters *b* and *c* on the basis of Popovics et al. [64] model to simulate curve softening and hardening, making the stress–strain model of FRP–stirrup-confined concrete extensive and universal. The relationship *f*(*x*) for the stress of confined concrete (*f_c_*) and the peak stress of confined concrete (*f_cc_*) is shown in Equation (9):

(9) $$f(x)=\frac{f_{c}}{f_{cc}}=\frac{x\cdot a}{a-1+{\left(x\right)}^{a{\left(x+\delta \right)}^{b}+c}}$$

(10) $$x=\frac{\varepsilon_{c}}{\varepsilon_{cc}}$$

(11) $$a=\frac{E_{c}}{E_{c}-E_{\sec}}=\frac{4730\sqrt{f_{co}}}{4730\sqrt{f_{co}}-f_{cc}/\varepsilon_{co}}$$

(12) $$c=\frac{\ln\left(\frac{f_{cc}\varepsilon_{cu}\cdot a}{f_{cu}\varepsilon_{cc}}-a+1\right)}{\ln\varepsilon_{cu}-\ln\varepsilon_{cc}}-a{\left(\frac{\varepsilon_{cu}}{\varepsilon_{cc}}+\delta \right)}^{-0.1}$$

where *E_sec_* is the secant modulus at the peak point; *E_c_* is the elastic modulus of the concrete; *f_co_* and *ε_co_* are the peak stress and corresponding strain of plain concrete, respectively; *f_c_* and *ε_c_* are the stress and strain of specimen, respectively; *f_cc_* and *ε_cc_* are the peak stress and peak strain of the specimen, respectively; and *f_cu_* and *ε_cu_* are the ultimate stress and ultimate strain of the specimen, respectively. For FRP- or FRP–stirrup-confined concrete columns, *b* and *δ* are −0.1 and 0.01, respectively. For stirrup-only confined concrete columns, both *b* and *δ* are 0.

**Figure 11:**
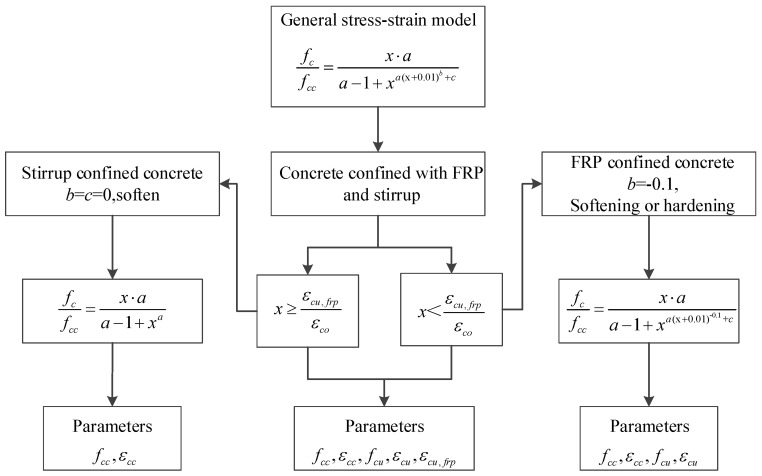
Flowchart for the application of the general model.

Figure 11 shows the flowchart for the application of the general model. The stress–strain relationship curve of stirrup-only confined concrete under axial compression (left branch in the flowchart) is only controlled by parameter *a*. The stress–strain relationship curve can be determined by the peak stress *f_cc_* and peak strain *ε_cc_* of the specimen. In the FRP-only confined concrete model (right branch in the flowchart), the curve before the peak point is determined by parameter *a*, while parameter *c* controls the strengthening or softening of the curve after the peak point. The stress–strain relationship curve can be determined by the peak stress *f_cc_*, peak strain *ε_cc_*, ultimate stress *f_cu_* and ultimate strain *ε_cu_* of the confined concrete column. For the FRP–stirrup composite-confined concrete columns (central branch in the flowchart), before FRP fracture, the stress–strain relationship curve of FRP–stirrup composite-confined concrete columns is similar to the stress–strain relationship curve of FRP-only confined concrete column. After the FRP fracture, the concrete is confined only by the stirrups, and the stress–strain relationship curve is similar to that of the stirrup-only confined concrete column.

### 5.3. Ultimate Stress f_cu_ and Ultimate Strain ε_cu_

The formulas for calculating *f_cu_* and *ε_cu_* of FRP–stirrup composite-confined circular concrete columns have been established [65]:

(13) $$\frac{f_{cu}}{f_{co}}=0.75+3.27{\left(\frac{f_{lf}}{f_{co}}\right)}^{0.9}+5.35\frac{{\left(f_{ls}\right)}^{0.86}}{f_{co}}\frac{A_{cc}}{A_{g}}$$

(14) $$\frac{\varepsilon_{cu}}{\varepsilon_{co}}=1.75+140\left(\frac{f_{lf}}{f_{co}}\right)\varepsilon_{fu}{}^{0.6}+20.6\frac{f_{ls}}{f_{co}}\frac{A_{cc}}{A_{g}}$$

The influence of the cross-section shape has not been considered in the above models, so the models for FRP–stirrup composite-confined concrete columns with different cross-section shapes should be further studied. Because the specimens studied are confined not only by the FRP but also by the stirrups, the effective confinement factor *k_e_* of the stirrups is introduced for the ultimate stress model of columns with noncircular sections. Considering the different cross-sectional shapes of the specimens, the section shape factor *k_s_* of FRP, the equivalent circular diameter *D* of the rectangular section and the core equivalent circular diameter *D_c_* of rectangular concrete in the core area are introduced. As shown in Figure 1, for the square section specimens, the red circle represents the equivalent circle of the square section; *D* is the equivalent circle diameter of the square section; *b* and *d* are the length and width of the square section, respectively; the blue circle represents the equivalent circle of square core concrete; *D_c_* is the equivalent circle radius of square core concrete; *b_c_* and *d_c_* are divided into length and width of square core concrete; *D* = *b* = *d*, *D_c_* = *b_c_* = *d_c_*. Then, the effective lateral confinement force of FRP–stirrup-confined concrete columns is calculated. Furthermore, considering the reduction in the confinement effect of the core concrete due to the smaller corner radius of the specimen section, it is suggested that the bearing capacity calculation model of FRP–stirrup composite-confined concrete columns with different corner radii (making the cross-section gradually change from square to circular) is as follows:

(15) $$\frac{f_{cu}}{f_{co}}=0.75+3.27\left(\left(1-\alpha \right)\frac{2R}{D}+\alpha \right){\left(\frac{k_{s}f_{lf}}{f_{co}}\right)}^{0.9}+5.35\left(\left(1-\alpha \right)\frac{2r}{D_{c}}+\alpha \right)\frac{{\left(k_{e}f_{ls}\right)}^{0.86}}{f_{co}}\frac{A_{cc}}{A_{g}}$$

where *R* is the corner radius of the outer FRP, *r* is the corner radius of the stirrups, *f_lf_* is the effective lateral restraint force of the FRP, *f_ls_* is the effective lateral restraint force of the spiral stirrups, *A_g_* is the total section area of the restrained concrete specimens and *A_cc_* is the core area of the restrained concrete with a stirrup.

To make the formula universal to specimens with different corner radii, the influence factor of the corner radius of section *α* is added into the above formula. Through the regression analysis of the database data, it is suggested that the value of *α* is 0.76.

To evaluate the accuracy and versatility of each model listed below, three major indicators (mean value AV, standard deviation SD and average absolute error AAE) were used as verification indicators to evaluate each model.

**Figure 12:**
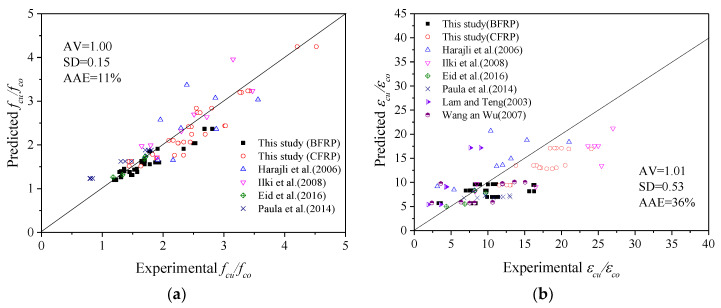
Comparison of the predicted results and test results of the ultimate point models: (**a**) ultimate stress; (**b**) ultimate strain.

The ultimate stress model takes full account of the influence of the cross-sectional shapes and corner radius of the specimens. The comparison of the prediction results and test results of the ultimate stress model is shown in Figure 12a. The calculation results of FRP-only, stirrup-only and FRP–stirrup-confined concrete columns with different cross-sectional shapes (circular, square and square with rounded corners) are in good agreement with the test results, especially those with smaller corner radii. The AV, SD and AAE values are 1.00, 0.15 and 11%, respectively. In conclusion, the ultimate stress models proposed above are applicable to FRP–stirrup composite-confined concrete columns with different section corner radii (making the cross-section gradually change from square to circular).

In addition, it was found that the ultimate strain model of FRP–stirrup composite-confined circular concrete columns performed well in predicting the data in this paper and in the database. As presented in Figure 12b, the AV, SD and AAE are 1.01, 0.53 and 36%, respectively, for the ultimate strain model. Based on the above analysis, the ultimate strain model of the circular columns (Equation (14)) has good versatility and can also be applied to predict the ultimate strain of FRP–stirrup composite-confined concrete columns with different cross-sectional shapes (circular, square and square with rounded corners).

### 5.4. Peak Stress f_cc_ and Peak Strain ε_cc_

The peak points (*f_cc_*, *ε_cc_*) of the stress–strain relationship curve of FRP–stirrup composite-confined concrete columns under axial compression are the turning points of the rising branch of the curves, which are between the linear elastic rising stage and the strengthening stage. The specific method of determining a peak point is to temporarily select a peak point on the actual test stress–strain relationship curve of FRP–stirrup composite-confined concrete columns and use the formula to determine the values of parameters *a* and *c* to obtain the theoretical stress–strain relationship curve. We then adjust the position of the peak point until the error between the theoretical stress–strain relationship curve and the test curve is minimised.

The peak stress and strain models of FRP–stirrup composite-confined circular concrete columns were obtained [65]. *β* is the peak strain inhibition coefficient of FRP to stirrup-confined concrete, and its recommended value is 0.65.

(16) $$\frac{f_{cc}}{f_{co}}=1+0.0015\frac{E_{l}}{f_{co}{}^{0.5}}+5.35\frac{f_{ls}{}^{0.86}}{f_{co}}\times \frac{A_{cc}}{A_{g}}$$

(17) $$\frac{\varepsilon_{cc}}{\varepsilon_{co}}=1+0.003\frac{E_{l}}{f_{co}{}^{0.5}}+\beta \cdot (1-0.002\frac{E_{l}}{f_{co}{}^{0.5}})20.6\cdot \frac{f_{ls}}{f_{co}}\cdot \frac{A_{cc}}{A_{g}}$$

Based on the above peak stress model for circular columns, the peak stress model of FRP–stirrup composite-confined concrete columns considering the FRP and stirrup lateral restraint efficiency and the interference of the section corner radius on the restraint efficiency of the core concrete is proposed.

(18) $$\frac{f_{cc}}{f_{co}}=1+0.0015\left(\left(1-\gamma \right)\frac{2R}{D}+\gamma \right)\frac{k_{s}E_{lf}}{f_{co}{}^{0.5}}+5.35\left(\left(1-\gamma \right)\frac{2r}{D_{c}}+\gamma \right)\frac{{\left(k_{e}f_{ls}\right)}^{0.86}}{f_{co}}\frac{A_{cc}}{A_{g}}$$

where *R* is the corner radius of the outer FRP, *r* is the corner radius of the stirrups, *E_lf_* is the circumferential stiffness of the FRP, *f_ls_* is the effective lateral restraint force of the spiral stirrups, *A_g_* is the total section area of the restrained concrete specimens and *A_cc_* is the core area of the restrained concrete with stirrups.

To make the formula universally applicable to specimens with different corner radii, the influence factor of the corner radius of section *γ* was added to the above formula. Through the regression analysis of the database data whose peak stress increase ratios *f_cc_*/*f_co_* were greater than 1, it is suggested that the value of *γ* is 0.55.

Considering the actual lateral restraint strength of specimens with different cross-sectional shapes except for a circular cross-section, the FRP stiffness and lateral restraint force in the peak strain model of FRP–stirrup composite-confined circular concrete are effectively reduced. It is suggested that the peak strain model of FRP–stirrup composite-confined concrete columns with different corner radii (making the cross-section gradually change from square to circular) is as follows:

(19) $$\frac{\varepsilon_{cc}}{\varepsilon_{co}}=1+0.003\frac{k_{s}E_{lf}}{f_{co}{}^{0.5}}+\beta \cdot \left(1-0.002\frac{k_{s}E_{lf}}{f_{co}{}^{0.5}}\right)\cdot 20.6\frac{k_{e}f_{ls}}{f_{co}}\frac{A_{cc}}{A_{g}}$$

**Figure 13:**
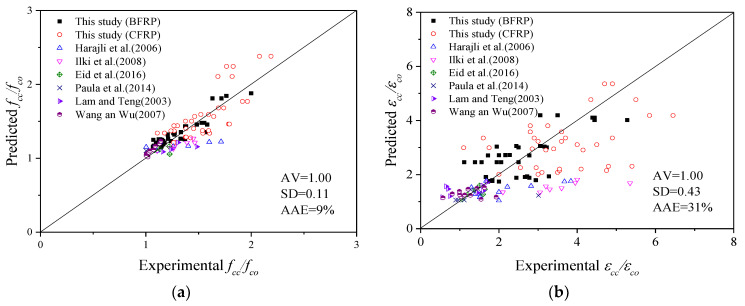
Comparison of the predicted results and test results of the peak point models: (**a**) Peak stress, (**b**) Peak strain.

A comparison of the prediction results and test results of the peak stress and strain models is shown in Figure 13. The peak point models can better predict the specimens in this database. The AV, SD and AAE of the ratio of the peak stress model are 1.00, 0.11 and 9%, respectively. The AV, SD and AAE values of the ratio of the peak strain model are 1.00, 0.43 and 9%, respectively. In conclusion, the peak stress and strain models proposed above are applicable to FRP–stirrup composite-confined concrete columns with different section corner radii (making the cross-section gradually change from square to circular).

### 5.5. Model Performance

**Figure 14:**
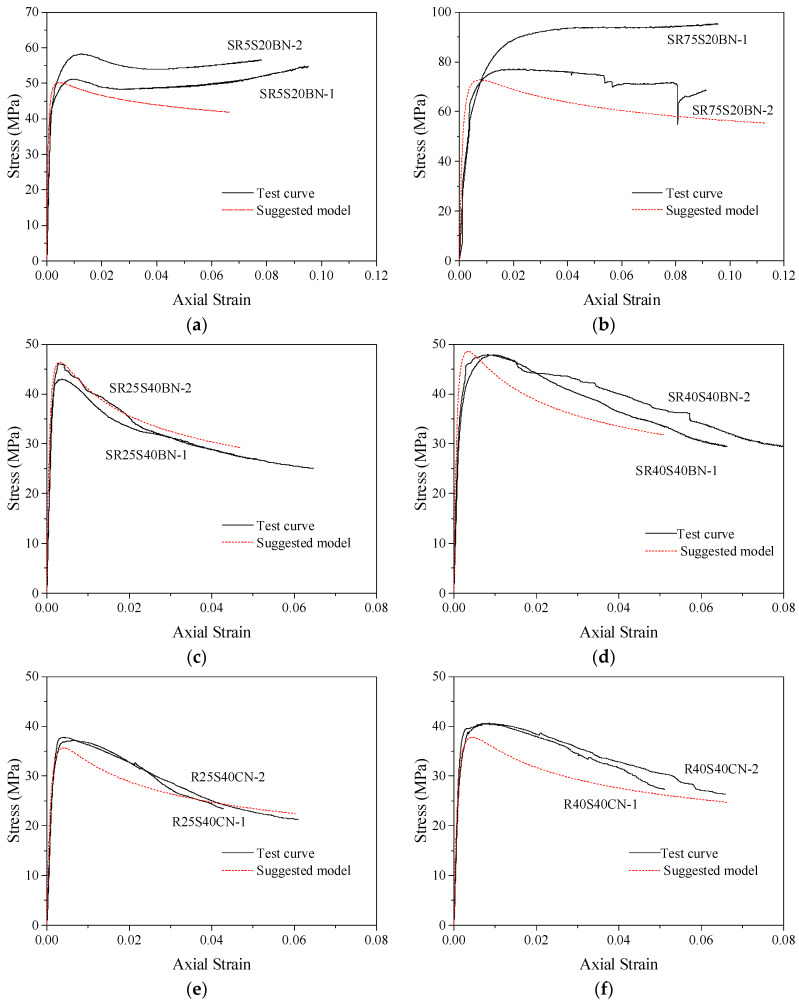

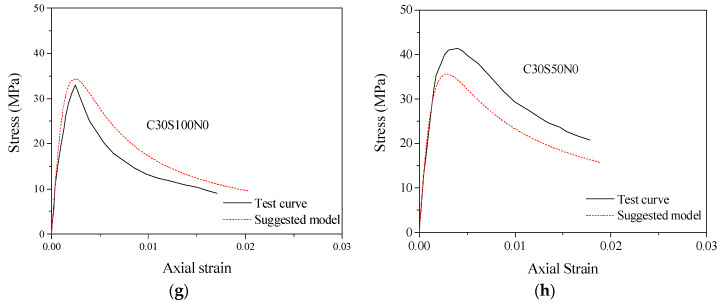
Performance of the stress–strain relationship curve model of stirrup-confined concrete columns: (**a**) SR5S20BN; (**b**) SR75S20BN; (**c**) SR25S40BN; (**d**) SR40S40BN; (**e**) R25S40CN; (**f**) R40S40CN; (**g**) C30S100N0; (**h**) C30S50N0.

**Figure 15:**
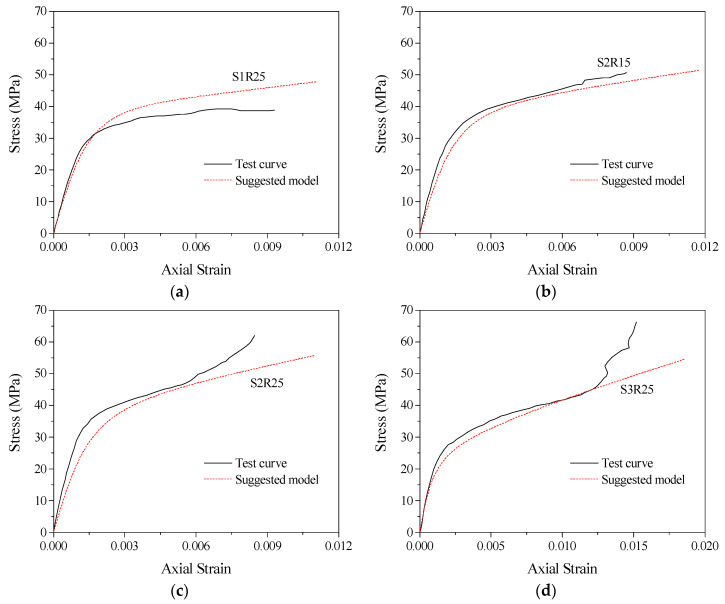
Performance of the stress–strain relationship curve model of FRP-confined concrete columns: (**a**) S1R25; (**b**) S2R15; (**c**) S2R25; (**d**) S3R25.

The general model proposed in this paper can predict the stress–strain relationship curves of concrete columns with different corner radii (making the cross-section gradually change from square to circular) confined by stirrups, FRP and combinations of the two. The accuracy of the stress–strain model can be judged by comparing the predicted curves with the test curves. The specific operation is to substitute the theoretical values of peak stress *f_cc_*, peak strain *ε_cc_*, ultimate stress *f_cu_* and ultimate strain *ε_cu_* of confined concrete columns into Equations (10) and (11) to determine the values of parameters *a* and *c*, thus obtaining the predicted stress–strain relationship curves of confined concrete column specimens in this database. Figure 14 compares the model prediction curve with the actual test curve of partially stirrup-only confined concrete columns studied by this test and others in the database. For example, Figure 14a SR5S20BN and Figure 14b SR75S20BN were studied in this test; the C30S100N0 and C30S50N0 specimens were studied by Eid et al. [59]. The section shapes of these specimens include round, square and square with corner rounding treatment. The comparison shows that the predicted curves of stirrup-only confined concrete columns are in good agreement with the test curves of specimens with larger stirrup spacing. In addition, the stirrup-only confined concrete column model is also applicable to other research specimens. Because FRP-only confined concrete columns were not tested in this paper, the accuracy of this model was evaluated by using data for FRP-confined concrete specimens with different corner radii from Lam and Teng [55] that are in the database. The comparison between the predicted stress–strain curves and test curves of concrete specimens S1R25, S2R15, S2R25 and S3R25 are shown in Figure 15. The results show that the proposed model can accurately predict the stress–strain relationship curves of FRP-confined concrete column specimens and better reflect the hardening section of the later curve.

**Figure 16:**
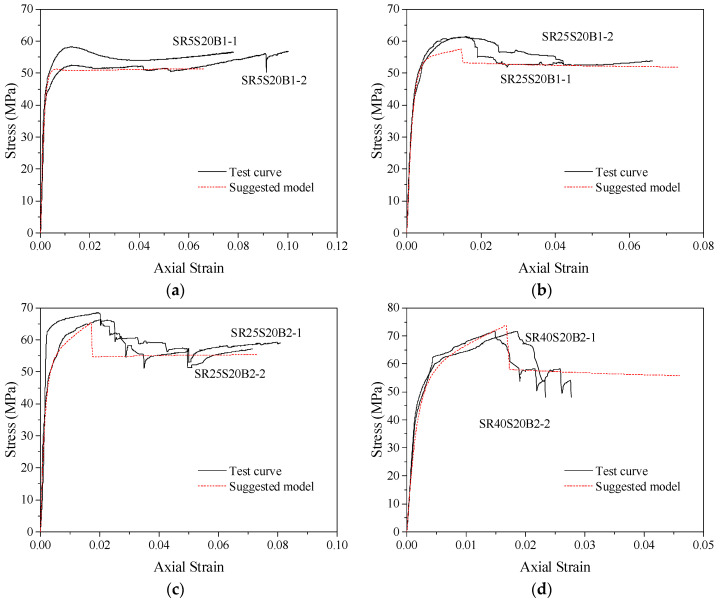

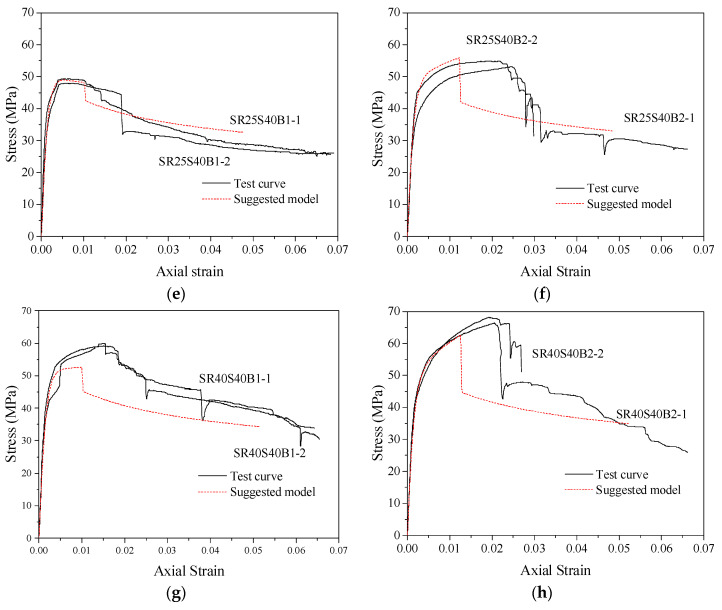
Performance of the stress–strain relationship curve model of FRP–stirrup composite-confined concrete: (**a**) SR5S20B1; (**b**) SR25S20B; (**c**) SR25S20B2; (**d**) SR40S20B2; (**e**) SR25S40B1; (**f**) SR25S40B; (**g**) SR40S40B1; (**h**) SR40S40B2.

**Figure 17:**
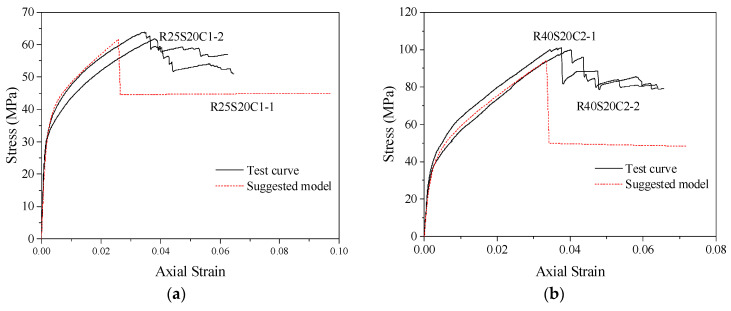

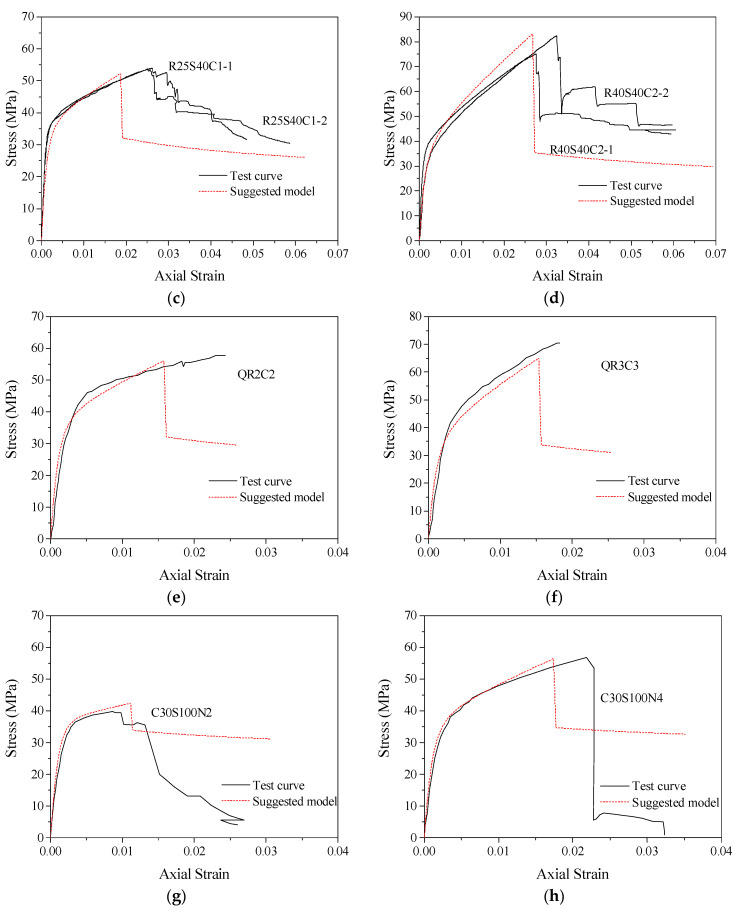

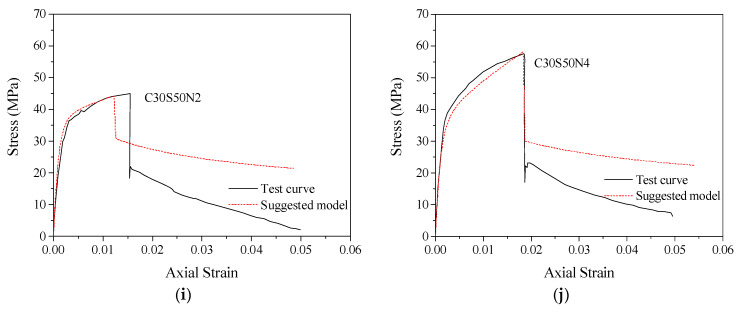
Performance of stress–strain relationship curve model of FRP–stirrup composite-confined concrete in the database: (**a**) R25S20C1; (**b**) R40S20C2; (**c**) R25S40C1; (**d**) R40S20C2; (**e**) QR2C2; (**f**) QR3C3; (**g**) C30S100N2; (**h**) C30S100N4; (**i**) C30S50N2; (**j**) C30S50N4.

The stress–strain relationship curve model of FRP–stirrup-confined concrete columns with different corner radii was analysed and evaluated by comparing test specimens and other research specimens in the database. The section shapes of these specimens include circular, square and square with rounded corners. The comparison between the model predicted curves and the test curves are shown in Figure 16 and Figure 17. The results show that the proposed model can completely simulate the stress–strain relationship curves of FRP–stirrup composite-confined concrete columns with different cross-sectional shapes (circular, square and square with rounded corners) in the elastic deformation stage, transition stage, strengthening stage and residual stage. Compared with the actual test curves, the predicted curves are accurate in predicting the elastic deformation stage. For the elastic–plastic deformation of the transition stage specimens, the hardening performance of the hardening stage curves is roughly fitted.

The predicted curves of the general model of confined concrete columns with different cross-sectional shapes (circular, square and square with rounded corners) not only coincide very well with the curves of the specimens tested in this paper, but they can also fully predict the specimens reported by others that are in the database. The model has a wide range of applicability, and its accuracy can provide a reference for the research of confined concrete structures with circular, square and square structures with rounded corner cross-sections. According to this, the foundation for practical engineering applications is laid.

## 6. Conclusions

Monotonic axial compression tests of 72 FRP–stirrup composite-confined concrete columns with different cross-sectional shapes (circular, square and square with rounded corners) were carried out. The test data of FRP-only and stirrup-only confined concrete columns are also summarised, the existing models of FRP–stirrup composite-confined concrete are contrasted, and the following conclusions are drawn:

(1) The cross-sectional shape directly affects the confinement efficiency of confined concrete columns. Compared with the circular section, the lateral confinement forces of confined rectangular section concrete columns are unevenly distributed around the section. The confinement efficiency of rectangular concrete columns is worse than that of circular concrete columns.
(2) For the FRP–stirrup composite-confined concrete columns with a small corner radius, FRP tensile fractures occur at the corners of the columns. For the specimens with a large corner radius, the FRP exhibited tensile failure or delamination; tensile failure occurred not only at the corners but also at the sides of the concrete columns.
(3) The larger the corner radius is, the closer the section approaches the circular section, the more uniform the lateral expansion deformation of concrete is and the more effective the lateral restraint provided by the FRP.
(4) The larger the corner radius is, the greater the slope and stiffness of the specimen in the strengthening stage; the more backwards the peak points and the ultimate points on the curves are; the stronger the ultimate bearing capacity and deformation capacity values of the specimens, especially for the specimens with a corner radius of 75 mm (a circular section).
(5) By introducing the effective confinement coefficient and cross-sectional shape coefficient of FRP and further considering the effect of a smaller corner radius on the confinement of core concrete columns, the peak points, ultimate points and stress–strain curve models are applicable to FRP, stirrup and FRP–stirrup-confined concrete columns with different cross-sectional shapes (circular, square and square with rounded corners)proposed, which was verified by a large number of confined concrete columns.

## Figures and Tables

**Table 1:** Summary of test results.

Specimen	*b*/*h* (mm)	*R* (mm)	FRP					Stirrup			*f_co_* (MPa)	*ε_co_* (%)	*f_cc_* (MPa)	*ε_cc_* (%)	*f_cu_* (MPa)	*ε_cu_* (%)
			Type	*t* (mm)	*k_s_*	*f_lf,e_*	*φ* (mm)	*S* (mm)	*k_e_*	*f_ls,e_*						
PC-BC1	150/150	75	/	/	/	0	/	/	/	0	/	/	40.48	0.0017	/	/
PC-BC2	150/150	75	/	/	/	0	/	/	/	0	/	/	38.59	0.0016	/	/
PC-BC3	150/150	75	/	/	/	0	/	/	/	0	/	//	31.59	0.0013	/	/
PC-BS1	150/150	0	/	/	/	0	/	/	/	0	/	/	40.66	0.0016	/	/
PC-BS2	150/150	0	/	/	/	0	/	/	/	0	/	/	40.59	0.0018	/	/
PC-BS3	150/150	0	/	/	/	0	/	/	/	0	/	/	39.27	0.0020	/	/
SR5S20BN-1	150/150	5	B	/	/	0	8	20	0.43	4.93	40.17	0.18	43.04	0.20	51.54	/
SR5S20BN-2	150/150	5	B	/	/	0	8	20	0.43	4.93	40.17	0.18	46.35	0.25	58.29	/
SR5S20B1-1	150/150	5	B	0.167	0.42	1.53	8	20	0.43	4.93	40.17	0.18	45.08	0.35	52.46	1.26
SR5S20B1-2	150/150	5	B	0.167	0.42	1.53	8	20	0.43	4.93	40.17	0.18	45.67	0.28	52.60	1.36
SR5S20B2-1	150/150	5	B	0.334	0.42	3.06	8	20	0.43	4.93	40.17	0.18	48.66	0.45	58.08	1.61
SR5S20B2-2	150/150	5	B	0.334	0.42	3.06	8	20	0.43	4.93	40.17	0.18	53.44	0.37	63.22	1.57
SR25S20BN-1	150/150	25	B	/	/	0	8	20	0.50	5.78	40.17	0.18	51.03	0.39	62.93	/
SR25S20BN-2	150/150	25	B	/	/	0	8	20	0.50	5.78	40.17	0.18	48.51	0.41	54.55	/
SR25S20B1-1	150/150	25	B	0.167	0.70	2.55	8	20	0.50	5.78	40.17	0.18	51.20	0.36	61.29	1.37
SR25S20B1-2	150/150	25	B	0.167	0.70	2.55	8	20	0.50	5.78	40.17	0.18	50.69	0.39	61.32	1.46
SR25S20B2-1	150/150	25	B	0.334	0.70	5.10	8	20	0.50	5.78	40.17	0.18	53.54	0.50	66.08	1.92
SR25S20B2-2	150/150	25	B	0.334	0.70	5.10	8	20	0.50	5.78	40.17	0.18	63.25	0.31	68.27	2.00
SR40S20BN-1	150/150	40	B	/	/	0	8	20	0.60	6.93	40.17	0.18	55.52	0.44	66.09	/
SR40S20BN-2	150/150	40	B	/	/	0	8	20	0.60	6.93	40.17	0.18	55.15	0.55	71.54	/
SR40S20B1-1	150/150	40	B	0.167	0.85	3.09	8	20	0.60	6.93	40.17	0.18	59.60	0.35	75.13	1.73
SR40S20B1-2	150/150	40	B	0.167	0.85	3.09	8	20	0.60	6.93	40.17	0.18	63.57	0.57	77.74	1.77
SR40S20B2-1	150/150	40	B	0.334	0.85	6.19	8	20	0.60	6.93	40.17	0.18	61.41	0.58	71.39	1.80
SR40S20B2-2	150/150	40	B	0.334	0.85	6.19	8	20	0.60	6.93	40.17	0.18	62.42	0.45	71.66	1.48
SR75S20BN-1	150/150	75	B	/	/	0	8	20	0.96	11.03	40.17	0.18	65.56	0.63	93.87	/
SR75S20BN-2	150/150	75	B	/	/	0	8	20	0.96	11.03	40.17	0.18	69.00	0.55	76.74	/
SR75S20B1-1	150/150	75	B	0.167	1	3.66	8	20	0.96	11.03	40.17	0.18	70.86	0.79	100.45	2.93
SR75S20B1-2	150/150	75	B	0.167	1	3.66	8	20	0.96	11.03	40.17	0.18	70.88	0.80	101.64	2.81
SR75S20B2-1	150/150	75	B	0.334	1	7.32	8	20	0.96	11.03	40.17	0.18	80.30	0.80	112.84	2.94
SR75S20B2-2	150/150	75	B	0.334	1	7.32	8	20	0.96	11.03	40.17	0.18	80.33	0.95	107.72	2.91
SR25S40BN-1	150/150	25	B	/	/	0	8	40	0.43	2.48	40.17	0.18	42.92	0.36	/	/
SR25S40BN-2	150/150	25	B	/	/	0	8	40	0.43	2.48	40.17	0.18	46.04	0.31	/	/
SR25S40B1-1	150/150	25	B	0.167	0.70	2.55	8	40	0.43	2.48	40.17	0.18	46.19	0.32	49.29	0.65
SR25S40B1-2	150/150	25	B	0.167	0.70	2.55	8	40	0.43	2.48	40.17	0.18	43.30	0.33	47.98	0.59
SR25S40B2-1	150/150	25	B	0.334	0.70	5.10	8	40	0.43	2.48	40.17	0.18	44.50	0.53	52.47	1.98
SR25S40B2-2	150/150	25	B	0.334	0.70	5.10	8	40	0.43	2.48	40.17	0.18	46.84	0.33	54.78	1.78
SR40S40BN-1	150/150	40	B	/	/	0	8	40	0.52	2.97	40.17	0.18	44.05	0.44	/	/
SR40S40BN-2	150/150	40	B	/	/	0.	8	40	0.52	2.97	40.17	0.18	47.32	0.50	/	/
SR40S40B1-1	150/150	40	B	0.167	0.85	3.09	8	40	0.52	2.97	40.17	0.18	46.85	0.48	59.96	1.50
SR40S40B1-2	150/150	40	B	0.167	0.85	3.09	8	40	0.52	2.97	40.17	0.18	49.73	0.30	59.15	1.39
SR40S40B2-1	150/150	40	B	0.334	0.85	6.19	8	40	0.52	2.97	40.17	0.18	55.02	0.49	66.36	2.05
SR40S40B2-2	150/150	40	B	0.334	0.85	6.19	8	40	0.52	2.97	40.17	0.18	55.15	0.59	68.10	1.90
PC-CC1	150/150	75	/	/	/	/	/	/	/	0	/	/	28.62	0.0023	/	/
PC-CC2	150/150	75	/	/	/	/	/	/	/	0	/	/	29.32	0.0021	/	/
PC-CC3	150/150	75	/	/	/	/	/	/	/	0	/	/	28.17	0.0022	/	/
PC-CS1	150/150	0	/	/	/	/	/	/	/	0	/	/	29.89	0.0020	/	/
PC-CS2	150/150	0	/	/	/	/	/	/	/	0	/	/	28.51	0.0017	/	/
PC-CS3	150/150	0	/	/	/	/	/	/	/	0	/	/	29.85	0.0023	/	/
SR5S20CN-1	150/150	5	C	/	/	0	8	20	0.43	4.93	29.42	0.20	34.62	0.35	48.09	/
SR5S20CN-2	150/150	5	C	/	/	0	8	20	0.43	4.93	29.42	0.20	32.62	0.22	42.40	/
SR5S20C1-1	150/150	5	C	0.167	0.42	4.07	8	20	0.43	4.93	29.42	0.20	37.32	0.57	55.60	5.73
SR5S20C1-2	150/150	5	C	0.167	0.42	4.07	8	20	0.43	4.93	29.42	0.20	36.44	0.68	56.02	5.40
SR5S20C2-1	150/150	5	C	0.334	0.42	8.15	8	20	0.43	4.93	29.42	0.20	40.62	0.83	68.81	8.28
SR5S20C2-2	150/150	5	C	0.334	0.42	8.15	8	20	0.43	4.93	29.42	0.20	42.77	0.64	71.92	7.16
SR25S20CN-1	150/150	25	C	/	/	0	8	20	0.50	5.78	29.42	0.20	38.24	0.32	42.35	/
SR25S20CN-2	150/150	25	C	/	/	0	8	20	0.50	5.78	29.42	0.20	37.27	0.58	43.60	/
SR25S20C1-1	150/150	25	C	0.167	0.70	6.78	8	20	0.50	5.78	29.42	0.20	40.49	0.72	61.60	3.86
SR25S20C1-2	150/150	25	C	0.167	0.70	6.78	8	20	0.50	5.78	29.42	0.20	42.18	0.60	63.73	3.43
SR25S20C2-1	150/150	25	C	0.334	0.70	13.55	8	20	0.50	5.78	29.42	0.20	43.06	0.90	75.23	3.86
SR25S20C2-2	150/150	25	C	0.334	0.70	13.55	8	20	0.50	5.78	29.42	0.20	48.38	0.80	76.72	3.81
SR40S20CN-1	150/150	40	C	/	/	0	8	20	0.60	6.93	29.42	0.20	46.94	0.65	68.74	/
SR40S20CN-2	150/150	40	C	/	/	0	8	20	0.60	6.93	29.42	0.20	44.78	0.56	64.37	/
SR40S20C1-1	150/150	40	C	0.167	0.85	8.23	8	20	0.60	6.93	29.42	0.20	51.16	0.56	88.68	3.44
SR40S20C1-2	150/150	40	C	0.167	0.85	8.23	8	20	0.60	6.93	29.42	0.20	49.68	0.72	89.07	3.40
SR40S20C2-1	150/150	40	C	0.334	0.85	16.45	8	20	0.60	6.93	29.42	0.20	57.79	0.74	100.70	3.69
SR40S20C2-2	150/150	40	C	0.334	0.85	16.45	8	20	0.60	6.93	29.42	0.20	56.40	0.98	99.83	4.00
SR75S20CN-1	150/150	75	C	/	/	0	8	20	0.96	11.03	29.42	0.20	53.52	0.94	79.21	/
SR75S20CN-2	150/150	75	C	/	/	0	8	20	0.96	11.03	29.42	0.20	49.54	0.98	72.64	/
SR75S20C1-1	150/150	75	C	0.167	1	9.73	8	20	0.96	11.03	29.42	0.20	53.80	1.10	96.82	3.75
SR75S20C1-2	150/150	75	C	0.167	1	9.73	8	20	0.96	11.03	29.42	0.20	51.96	0.87	96.00	3.60
SR75S20C2-1	150/150	75	C	0.334	1	19.46	8	20	0.96	11.03	29.42	0.20	61.14	1.17	123.72	4.20
SR75S20C2-2	150/150	75	C	0.334	1	19.46	8	20	0.96	11.03	29.42	0.20	64.37	1.29	132.95	4.81
SR25S40CN-1	150/150	25	C	/	/	0	8	40	0.43	2.48	29.42	0.20	37.80	0.40	/	/
SR25S40CN-2	150/150	25	C	/	/	0	8	40	0.43	2.48	29.42	0.20	37.14	0.60	/	/
SR25S40C1-1	150/150	25	C	0.167	0.70	6.78	8	40	0.43	2.48	29.42	0.20	41.91	0.62	53.74	2.62
SR25S40C1-2	150/150	25	C	0.167	0.70	6.78	8	40	0.43	2.48	29.42	0.20	41.94	0.70	53.72	2.51
SR25S40C2-1	150/150	25	C	0.334	0.70	13.55	8	40	0.43	2.48	29.42	0.20	47.22	0.71	72.46	3.32
SR25S40C2-2	150/150	25	C	0.334	0.70	13.55	8	40	0.43	2.48	29.42	0.20	45.35	0.95	71.13	4.10
SR40S40CN-1	150/150	40	C	/	/	0	8	40	0.52	2.97	29.42	0.20	40.46	0.71	/	/
SR40S40CN-2	150/150	40	C	/	/	0	8	40	0.52	2.97	29.42	0.20	40.64	0.82	/	/
SR40S40C1-1	150/150	40	C	0.167	0.85	8.23	8	40	0.52	2.97	29.42	0.20	45.25	0.59	66.15	2.60
SR40S40C1-2	150/150	40	C	0.167	0.85	8.23	8	40	0.52	2.97	29.42	0.20	46.95	0.51	66.04	2.31
SR40S40C2-1	150/150	40	C	0.334	0.85	16.45	8	40	0.52	2.97	29.42	0.20	52.50	0.96	74.90	2.76
SR40S40C2-2	150/150	40	C	0.334	0.85	16.45	8	40	0.52	2.97	29.42	0.20	52.75	1.08	82.36	3.24

**Table 2:** Summary of existing FRP and stirrup-confined rectangular concrete models.

Model	Ultimate Stress	Ultimate Strain	Stress–Strain Relationship
Hariajil et al. [56,57] model	$\frac{f_{cu}}{f_{co}}=1+1.25{\left(\frac{f_{lf,e}+f_{ls,e}\left(A_{cc}/A_{g}\right)}{f_{co}}\right)}^{-0.5}$	$\frac{\varepsilon_{cu}}{\varepsilon_{co}}=\left[\left(\frac{25800e^{1.17b/h}}{{\left(\rho_{f}E_{f}\right)}^{0.83}}\right)\varepsilon_{l}+2.0\right]\left(\frac{f_{cu}}{f_{co}}-1\right)$	$\left\{ \begin{array}{l} f_{c}=f_{o}\left[\frac{2\varepsilon_{c}}{\varepsilon_{o}}-{\left(\frac{\varepsilon_{c}}{\varepsilon_{o}}\right)}^{2}\right]\\ f_{c}=\sqrt{\left(K_{o}^{2}-K\right)}-K_{o} \end{array}\right.$ $f_{o}=f_{co}+k\left[f_{lf,e}+f_{ls,e}\left(A_{cc}/A_{g}\right)\right]$ $\varepsilon_{o}=\varepsilon_{co}\left[1+\left(310.57\varepsilon_{lo}+1.9\right)\left(\frac{f_{o}}{f_{co}}-1\right)\right]$
Ilki et al. [58] model	${\left[\frac{f_{cu}}{f_{co}}-1\right]}_{TOTAL}={\left[\frac{f_{cu}}{f_{co}}-1\right]}_{FRP}+{\left[\frac{f_{cu}}{f_{co}}-1\right]}_{TSR}$ ${\left[\frac{f_{cu}}{f_{co}}\right]}_{FRP}=\left[1+2.54\left(\frac{f_{lf,e}}{f_{co}}\right)\right]$ ${\left[\frac{f_{cu}}{f_{co}}\right]}_{TSR}=\left[1+4.54\left(\frac{f_{ls,e}}{f_{co}}\right)\right]$	${\left[\frac{\varepsilon_{cu}}{\varepsilon_{co}}-1\right]}_{TOTAL}={\left[\frac{\varepsilon_{cu}}{\varepsilon_{co}}-1\right]}_{FRP}+{\left[\frac{\varepsilon_{cu}}{\varepsilon_{co}}-1\right]}_{TSR}$ ${\left[\frac{\varepsilon_{cu}}{\varepsilon_{co}}\right]}_{FRP}=\left[1+19.27\left(\frac{b}{h}\right){\left(\frac{f_{lf,e}}{f_{co}}\right)}^{0.53}\right]$ ${\left[\frac{\varepsilon_{cu}}{\varepsilon_{co}}\right]}_{TSR}=\left[1+5\left({\left(\frac{f_{cu}}{f_{co}}\right)}_{TSR}-1\right)\right]$	/
Pellegrino and Modena [59] model	$\frac{f_{cu}}{f_{co}}=1+k_{1}{\left(\frac{f_{l,e}}{f_{co}}\right)}^{1-\alpha }$ $k_{1}=k_{A}\cdot k_{R}$ $k_{A}=A{\left(\frac{f_{l,e}}{f_{co}}\right)}^{-\alpha }$ $\left\{ \begin{array}{l} k_{R}=1-2.5\left(0.3-2r/b\right)2r/b<0.3\\ k_{R}=12r/b\geq 0.3 \end{array}\right.$	$\frac{\varepsilon_{cu}}{\varepsilon_{co}}=2+B\frac{f_{l,e}}{f_{co}}$	$f_{c}=\frac{\left(E_{0}-E_{1}\right)\varepsilon_{c}}{{\left[1+{\left(\frac{\left(E_{0}-E_{1}\right)\varepsilon_{c}}{f_{1}}\right)}^{n}\right]}^{\frac{1}{n}}}+E_{1}\varepsilon_{c}$
Faustino et al. [60] model	$f_{cu}=f_{co}+3.7\left(\frac{2R}{b}\right)f_{l}$ $f_{l}=f_{lf}+f_{ls}=\frac{2A_{sp}}{d_{s}\cdot s}f_{hy}+\frac{4n_{f}t_{f}}{d}E_{f}\varepsilon_{l}$	$\varepsilon_{cu}=18.89\varepsilon_{co}\left(\frac{f_{l}}{f_{co}}\right)$	$f_{c}=\frac{\left(E_{0}-E_{1}\right)\varepsilon_{c}}{{\left[1+{\left(\frac{\left(E_{0}-E_{1}\right)\varepsilon_{c}}{f_{1}}\right)}^{n}\right]}^{\frac{1}{n}}}+E_{1}\varepsilon_{c}\leq f_{cu}$
Eid et al. [61] model	$\frac{f_{cu}}{f_{co}}=1+3.3\left(\frac{f_{ls,e}}{f_{co}}+\xi \frac{f_{lf,e}}{f_{co}}\right)\geq \frac{f_{cc}}{f_{co}}$	$\frac{\varepsilon_{cu}}{\varepsilon_{co}}=1.56+12\left(\frac{f_{ls,e}}{f_{co}}+\xi \frac{f_{lf,e}}{f_{co}}\right){\left(\xi \frac{\varepsilon_{f}}{\varepsilon_{co}}\right)}^{0.45}\geq \frac{\varepsilon_{cc}}{\varepsilon_{co}}$	$f_{c}=\frac{a\varepsilon_{c}}{1+b\varepsilon_{c}+z\varepsilon_{c}^{2}}$

**Table 3:** Constraint efficiency of constrained rectangular concrete column specimens.

Researchers	Number	*b*/*h*	*R* (mm)	*S* (mm)	*f_co_* (MPa)	*f_lf_*/*f_co_*	*f_ls_*/*f_co_*
This study	72	1	5–75	20–40	29.42–40.17	0–0.66	0.06–0.37
Wang and Wu [47]	12	1	0–75	/	30.7–32.3	0.10~0.62	0
Lam and Teng [55]	12	1–1.5	15–25	/	24–41.5	0.17–1.16	0
Harajli et al. [56]	12	1–2.7	15	100	15.2	0–0.96	0–0.02
Ilki et al. [58]	8	1–2	10–40	175–200	10.83–23.44	0.29–1.14	0.01–0.02
Eid et al. [61]	6	1	15	50–100	33.7	0–0.30	0.01–0.03
Paula et al. [62]	12	1	0–38	100	34.6	0–0.35	0.01

## Data Availability

The data presented in this study are available on request from the corresponding author.
